# Normal Aging Affects the Short-Term Temporal Stability of Implicit, But Not Explicit, Motor Learning following Visuomotor Adaptation

**DOI:** 10.1523/ENEURO.0527-20.2021

**Published:** 2021-10-13

**Authors:** Guneet Bindra, Rylee Brower, Ryan North, Weiwei Zhou, Wilsaan M. Joiner

**Affiliations:** 1 Department of Neurobiology, Physiology and Behavior, University of California, Davis, Davis, California 95616; 2 Department of Neurology, University of California, Davis, Davis, California 95616

**Keywords:** aging, explicit, implicit, motor adaptation, retention, temporal

## Abstract

Normal aging is associated with a decline in memory and motor learning ability. However, the exact form of these impairments (e.g., the short-term temporal stability and affected learning mechanisms) is largely unknown. Here, we used a sensorimotor adaptation task to examine changes in the temporal stability of two forms of learning (explicit and implicit) because of normal aging. Healthy young subjects (age range, 19–28 years; 20 individuals) and older human subjects (age range, 63–85 years; 19 individuals) made reaching movements in response to altered visual feedback. On each trial, subjects turned a rotation dial to select an explicit aiming direction. Once selected, the display was removed and subjects moved the cursor from the start position to the target. After initial training with the rotational feedback perturbation, subjects completed a series of probe trials at different delay periods to systematically assess the short-term retention of learning. For both groups, the explicit aiming showed no significant decrease over 1.5 min. However, this was not the case for implicit learning; the decay pattern was markedly different between groups. Older subjects showed a linear decrease of the implicit component of adaptation over time, while young subjects showed an exponential decay over the same period (time constant, 25.61 s). Although older subjects adapted at a similar rate, these results suggest natural aging selectively impacts the short-term (seconds to minutes) temporal stability of implicit motor learning mechanisms. This understanding may provide a means to dissociate natural aging memory impairments from deficits caused by brain disorders that progress with aging.

## Significance Statement

Although normal aging is known to reduce learning retention, the time course and specific mechanisms that underlie these deficits are largely unknown. Here, for young and older subjects, we examined the contribution of two learning mechanisms (implicit and explicit) to motor output adjustments in response to changes in visual feedback. We also systematically quantified the short-term temporal decay of these different types of learning in both groups. We found that over a short time period explicit movement planning was not significantly different between the two age groups, but the stability of implicit motor learning was affected by normal aging. Interestingly, older subjects showed a distinctly different pattern of decay, suggesting an age-dependent impairment in the short-term retention of implicit motor learning.

## Introduction

Normal aging is consistently associated with learning and memory deficits (Seidler et al., 2010; King et al., 2013; Cai et al., 2014), which accompany impaired decision-making and motor performance (Cai et al., 2014). For example, young and older subjects can learn sequences in a serial reaction time task, but older subjects demonstrate reduced performance when learning probabilistic sequences (Dennis et al., 2006; Clark et al., 2015). In addition, younger subjects perform significantly better at pairing items in associative binding tasks (Light et al., 2004; Provyn et al., 2007; Old and Naveh-Benjamin, 2008). Aging also correlates with memory deficits (Nyberg et al., 2012; King et al., 2013); working memory declines with age (Park et al., 2002; Malouin et al., 2010), along with forms of long-term memory (e.g., episodic; Rönnlund et al., 2005; Metzler-Baddeley et al., 2011; Sauzéon et al., 2016). However, studies have also suggested that other types of memory are preserved during aging (e.g., semantic memory; Spaniol et al., 2006; St-Laurent et al., 2011; Kennedy et al., 2015).

Within the context of movement, associations between aging and reduced motor adaptation have been shown (Wolpe et al., 2020). Trewartha et al. (2014) found that despite similar adjustments to physical perturbations of arm reaching movements, older subjects demonstrated less retention and poor explicit memory performance compared with young subjects. Likewise, Malone and Bastian (2016) found that when learning a novel walking pattern, older subjects were more likely to forget and show a performance reduction after breaks. However, despite the known effect of aging on learning retention, the exact temporal properties (e.g., short-term temporal stability) of this deficit are largely unknown.

Prior studies have shown that short-term motor adaptation involves concurrent learning mechanisms with different temporal properties and drivers (i.e., error signal sensitivity; Smith et al., 2006; Taylor et al., 2014). Previous studies have also demonstrated the temporal decay of the memory trace in a wide range of tasks, suggesting that motor adaptation provides a tractable method to probe both different learning mechanisms and changes in relative stability (Sing et al., 2009; Talamini and Gorree, 2012; Hadjiosif and Smith, 2013; Kitago et al., 2013; Loaiza and McCabe, 2013; Taylor et al., 2014; Zhou et al., 2017). One notable task involves visuomotor rotation (VMR), in which individuals adapt to a visual feedback perturbation (i.e., a rotation of the feedback trajectory; Krakauer et al., 1999; Redding et al., 2005). This adaptation has been shown to involve concurrent explicit and implicit learning mechanisms (Taylor et al., 2014). Implicit learning mechanisms use sensory prediction errors to update the internal forward model, likely mediated by the cerebellum on a subconscious level (Tseng et al., 2007; Synofzik et al., 2008). Additionally, explicit processes also contribute to the adaptation to the rotation of the feedback through conscious efforts, minimizing the experienced target errors (Mazzoni and Krakauer, 2006; Bond and Taylor, 2015). Thus, the VMR paradigm provides a method for assessing both explicit and implicit components of motor adaptation; the explicit component of learning is determined through an aiming report made by the subject, while the implicit learning is based on subtracting this planned movement direction from the actual movement trajectory (Taylor et al., 2014; Morehead et al., 2015). Furthermore, studies have used this VMR task to examine age-dependent differences in learning. Bock (2005) and Buch (2003) used this paradigm to measure age-related variations in adaptation. However, these learning differences were either inferred by different methods (i.e., a combination of motor and nonmotor tests) or were based on the size of motor adaptation aftereffects. More recent studies have suggested that aging specifically reduces the extent to which explicit learning mechanisms are used during training to adjust motor output through more direct measures (Vandevoorde and Orban de Xivry, 2019) or are inferred through neuroimaging and nonmotor memory tests (Wolpe et al., 2020).

Based on the studies above, there are age-related differences in the use of concurrent learning mechanisms during motor adaptation. However, age-based variations in the short-term (seconds to minutes) temporal properties of these mechanisms (i.e., stability of the memory trace) remain uncertain. Here, we adapted a previous VMR adaptation paradigm (Hadjiosif and Smith, 2013; Zhou et al., 2017) to directly assess the extent to which normal aging affects the use of different learning mechanisms and then systematically examined their relative temporal stability over 1.5 min. Based on the studies above, we hypothesized that older subjects would demonstrate a reduced short-term temporal stability of the overall adaptation, and we focused on quantifying the influence of age on the rate of decrease and identifying the driver (implicit or explicit learning mechanisms) of this decay process.

## Materials and Methods

### Participants

Twenty young subjects (10 women; age range, 19–28 years) and 19 older subjects (14 women; age range, 63–85 years) without known neurologic impairment were recruited from the University of California, Davis, and the surrounding community to participate in the study. The cognitive function of all subjects was evaluated by using the Mini-Mental State Exam (MMSE; Folstein et al., 1975) and Trail Making Test (TMT, parts A and B; Army Individual Test Battery, 1944; Reitan and Wolfson, 1985). Scores on both the MMSE and the TMT did not reach the cutoff scores for concerns of cognitive impairment (24 of 30 on MMSE, 78 s for TMT part A, 273 s for part B) and were not significantly different between groups. Handedness of the subjects was measured by the Edinburgh Handedness Inventory (Oldfield, 1971). All young subjects recruited were right handed, and 2 of 19 older subjects were left handed. Note that performance for these two subjects was not different from that of the respective group. All subjects gave written informed consent and received financial compensation for their participation. The study protocol was approved by the University of California, Davis, Institutional Review Board.

### Experimental apparatus

**Figure 1.**
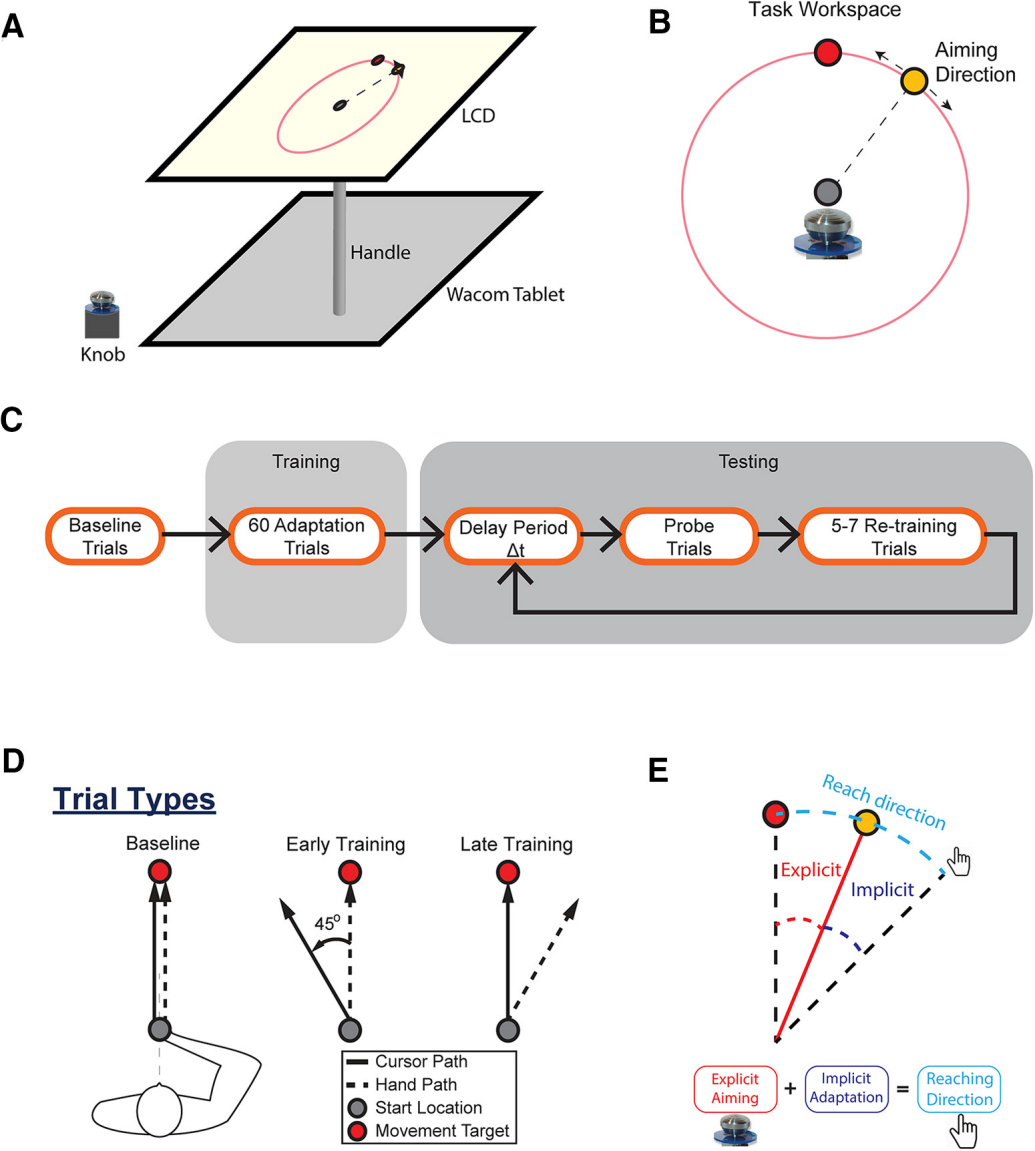
Experimental design and task structure. ***A***, The experimental setup consisted of an LCD monitor that displayed targets and visual feedback of the hand, a digitizing tablet, and a PC that controlled the paradigm. The monitor was 10 inches above the digitizing tablet. The midline of the subject was aligned with the center of the tablet and the monitor. ***B***, Depiction of the explicit aim selection, which consisted of a circle centered at the start location (gray filled circle). The movement goal is represented by the red filled circle, and a yellow filled circle represents the selection marker. This circle appeared 10 cm from the starting position. Subjects turned a knob at the beginning of each trial to move the yellow marker around the circle and indicate their explicit aiming direction. ***C***, The sequence of training and testing the subjects. After 20 familiarization trials, the subjects underwent 40 baseline trials where the cursor followed the hand position. This was followed by 60 training trials, during which a +45° (clockwise) or −45° (counterclockwise) visuomotor rotation of the cursor movement feedback was applied. During the testing phase, each subject experienced a delay period (0, 5, 10, 20 30, 50, and 90 s). This was followed by a single probe trial, and then 5-7 retraining trials before the next delay period was tested. This pattern was repeated for a total of 4 probe movements for each delay duration. ***D***, Depiction of hand and cursor movements during baseline, and early and late training. During baseline, the cursor and hand paths were aligned. When the visuomotor rotation was applied, there was a rotational offset between the cursor and hand path. Late in training, subjects adjusted the hand path to guide the cursor to the target. ***E***, A representation of the explicit and implicit components of overall adaptation. Explicit aiming was measured as the angle difference between the aiming direction and the target, while implicit adaptation was defined as the angle difference between reach direction and aiming direction.

In the task, subjects were seated at a desk in a dimly lit room facing a horizontal 27 inch LCD monitor (Fig. 1*A*). The chair height was adjusted for each subject so that they could comfortably perform the task and view the screen. The experimental system included a monitor, a digitizing tablet, and a PC to run the experimental paradigm and collect the behavioral data. The LCD monitor was mounted horizontally in front of subjects at shoulder level, displaying the various visual cues during the experiment. The monitor was 10 inches above the digitizing tablet (a workspace of 12 by 19 inches; Intuos4, Wacom) that tracked and recorded hand position at 200 Hz. Subjects grasped a cylindrical handle (diameter, 2.5 cm) containing the tablet stylus inside. The hand/stylus moved on the tablet below the monitor (refresh rate, 60 Hz), with its position presented on the above screen as a round cursor (diameter, 0.3 cm). The midline of the subject was aligned with the center of the tablet and monitor. This also served as the center of the workspace. The position of the LCD monitor obstructed the vision of the tablet and the arm movements made by subjects. The large 27 inch screen provided sufficient space to ensure that the space between the edge of the screen and visual objects used in the task (e.g., the circular path of the rotation marker) did not provide unintended spatial landmarks to the subjects. A rotation knob (diameter, 3.8 cm) was mounted on the left side of the tablet to allow the subjects to choose their aiming direction before each movement.

### Experimental paradigm

On each trial, the subject reached from a circular central target (8 mm in diameter) located at the center of the screen (20 cm from the body on the sagittal axis of the body) to a circular reach target (diameter, 10 mm) located 10 cm away from the starting position along the midline. At the start of the trial, a large circle (diameter, 10 cm) was displayed centered at the starting location, showing the path for the rotation maker and indicating the spatial relationship among the marker and targets. An orange rotation marker was shown on this circular path and could be moved along the path by rotating the knob (Fig. 1*B*). The subject was instructed to first turn the rotation knob with their left hand to select an aiming direction for the planned movement. Once the subject had made a selection by pressing the knob, the display of the circular path and rotation maker was removed. Subjects then could initiate the movement of the cursor from the center location to the reach target using their right hand (Movie 1, VMR Decay Task.mp4, examples of the task structure/requirements and the different trials used in the experiment). Subjects were instructed to move the cursor from the start position to the target as quickly and fluidly as possible. Subjects received visual and auditory feedback on the speed of their movement to ensure consistency of speed and duration. If the movement was too slow (duration, ≥400 ms), the target turned blue once the radial distance was exceeded. If the movement was too fast (≤200 ms), the target turned red. The target turned green for movement durations that were between 200 and 400 ms. In addition, a short-duration beep (frequency, 429 Hz) was provided to signify a good trial. Note that this feedback was based only on movement duration and not on movement accuracy. On adaptation trials, during the 10 cm point-to-point reaching movement full visual feedback of the cursor was provided to the subject. Once the movement exceeded the radial distance of the target (10 cm), only the end-point feedback (cursor at a location where the movement exceeded 10 cm) was provided. One second after the end-point feedback was presented, the cursor was removed and replaced by a large circle centered on the initial start location. The radius of the circle matched the distance of the hand/cursor from the start location. Subjects were guided to move their hand back to the start location by the size of the circle (the radius of the circle decreased proportionally to the distance between the hand/cursor and the start location), which limited the spatial information of the actual movements while providing enough information to guide the subject back to the start location. Once the hand/cursor was within 2 cm of the start location, the cursor reappeared, and the subject brought the cursor back to the central target. The intertrial interval was set to 1 s. At the beginning of each trial, the orange rotation marker appeared 10 cm above the central start position along the circular path.

Movie 1. VMR Decay Task.mp4. The video illustrates the visual display and objects controlled during the experiment: the selection marker, the circular path, target, and cursor. Three different trial examples are sequentially shown: (1) an early training trial, (2) a late training trial, and (3) a retention probe trial (5 s delay). 10.1523/ENEURO.0527-20.2021.video.1

All subjects in both groups (young and older group) experienced the same task structure used in previous studies (Fig. 1*C*; Hadjiosif and Smith, 2013; Zhou et al., 2017). Subjects began the task with 20 familiarization trials to ensure that they were accustomed to the experimental design and desired movement speed. During baseline (40 trials), the cursor followed the true position of the hand/stylus on the tablet below (Fig. 1*D*). On a random number of trials (20%) visual feedback was removed (blank trials). These blank trials (a total of eight blank trials) served as the baseline for the retention probe trials described below. After baseline, subjects were trained on a visuomotor rotation (60 trials) during which the cursor path was rotated around the hand path by either a +45° [clockwise (CW)] or −45° [counterclockwise (CCW)] perturbation. Each subject experienced only one type of rotation (CW or CCW), and the direction of the VMR was counterbalanced across all subjects. We defined three periods during training to compare learning between groups [early training: the first five training trials (1–5); middle training: the middle five trials of training (28–32); and late training: the last five training trials (56–60)]. Note that these periods were used to show the progression in behavior throughout training and were based on the total number of training trials. Also, the results are similar for different window sizes (three or seven trials).

After training, subjects completed the testing phase based on a task structure developed by Hadjiosif and Smith (2013). Subjects underwent a series of retention probe trials and retraining trials for seven different delay periods (0, 5, 10, 20, 30, 50, and 90 s). The sequence is depicted in Figure 1*C*. Throughout the delay, subjects held the cursor in the start target, which turned light green as a signal for the subject to maintain their cursor at the center position. After the delay period, the selection marker appeared on the circular path to allow the subject to select the planned movement direction of the upcoming arm movement. The display disappeared after the selection, cueing the subject to perform the reach. This retention probe trial, which was the first trial after the delay period ended, were also blank trials, meaning that there was no visual feedback. The probe trial for each delay period was followed by five to seven retraining trials during which subjects performed movements to the trained target with the rotation and full visual feedback. This pattern (one retention probe movement followed by five to seven retraining movements) was repeated throughout the session, for a total of four probe movements for each delay duration. The delays were randomly selected throughout the session. Because of the randomness of the number of retraining trials, subjects made a total of 168–224 movements divided between two blocks. The entire session (40 min for behavioral testing and 30 min for clinical evaluations—the Mini-Mental State Exam and Trail Making Test) was ∼1 h and 30 min with breaks between blocks. A short video illustrating an example of an early training trial, a late training trial, and a retention probe trial can be found in the video VMR Decay Task.mp4 (Movie 1).

### Analysis of the movement data

The movement duration was defined as the interval between the times when the hand velocity exceeded 0.05 m/s and when the hand crossed the 10 cm radius. We focused on the initial heading angle of the hand—the feedforward mechanisms involved in the movement plan. We determined the reach angle of each movement soon after initiation by computing the angle of the line connecting the hand position at 1 and 3 cm into the movement. To quantify the contributions of explicit and implicit learning to the motor recalibration in response to the rotated visual feedback, we performed a procedure that was similar to previous studies (Taylor et al., 2014; Bond and Taylor, 2015; Morehead et al., 2015). The explicit aiming was measured by the angles that subjects selected as their aiming direction (the angle of the line connecting the start location and rotation marker). The implicit learning was calculated by subtracting the aiming angles from the actual reaching angles (Fig. 1*E*). For simplicity, we refer to the target direction as 0° and classify all movements relative to this direction in the analysis. The experiment was designed to examine the retention of these two types of learning after short time delays. For each time delay, the retention of learning was quantified by comparing the angular deviation on the no-visual-feedback probe trials to the average angular deviation at the end of the preceding retraining trials (the last three trials of the five- to seven-trial retraining sequence). The absolute retention (difference in angular deviation between the probe trial and the average on the retraining trials) and relative retention (percentage of the adaptation retained: (probe trial angle – average angle on the retraining trials)/average angle on the retraining trials) were used to examine the decay of the two learning components and overall learning over the delay periods. These measures of retention were a direct comparison of before and after the delay; there was no need for a baseline correction. Because of the variance in the selection time and movement duration across subjects, we used the actual elapsed time to define the delay for explicit and implicit learning instead of the imposed seven delay periods in the experiments to better capture the influence of the delay for each learning component. Thus, the delay for the explicit learning component was defined as the time interval from the end of the previous trial to the time when the selection was made. The delay for the implicit learning component was defined as the time interval from the end of the previous trial to the movement onset. (Note that the actual elapsed time was always slightly longer for the implicit learning component.)

As in previous work (Zhou et al., 2017), we applied a standard exponential to determine the time constant of decay, as follows:

$$y=y_{0}\cdot e^{\frac{-t}{\tau }}+C.$$

Here, the amount of learning (*y*) over time, *t*, depends on (1) the scaling term, $y_{0}$; (2) the time constant, τ and (3) an offset term, *t*, that represents the amount of learning that remains stable following the short-term decay. We also fitted the data using an alternative linear model to determine which model provided a better description of the data, as follows:

y=k⋅t + C,

where k is the slope of the linear regression and *C* is the intercept. We fitted the data with the two models using functions *fitnlm* and *fitlm* in MATLAB and determined the goodness-of-fit by comparing the Akaike information criterion (AIC; Akaike, 1974) of the two models (a model is considered to be a better fit for the data if its AIC is smaller than the AIC of the other model).

In addition to determining the time course of explicit and implicit learning throughout training, and the relative stability of the memory traces following the elapsed time, we also examined the temporal properties of the selection process of the explicit movement plan. Through the rotation knob, we quantified the time required to select the explicit movement plan direction. The selection time for aiming was defined as the interval between when subjects started rotating the knob and when they pressed the knob. We examined the selection time of the explicit aiming process throughout the training trials and over the elapsed time.

### Statistical analysis

Data were analyzed offline using MATLAB 2019a (MathWorks) and R version 3.6.2 (r-project.org). Trials that had very slow/fast movement speeds (peak speed, <0.22 or >0.5 m/s) and long selection time (selection time, >30 s) were excluded (∼3% of total trials for the young group and ∼4.5% of total trials for the older group). The reason we excluded the trials with a selection time >30 s is that all these trials were at the beginning of the training session when subjects first experienced the visuomotor rotation and were becoming familiar with rotating the knob and making an aiming selection. There were 2.1% of training trials with a selection time >30 s for the older group and 0.6% for the young group. All of these trials were in the first half of the training phase; none of the subjects made a selection with a time >30 s for trials in the second half of the training session and the entire testing phase. We applied a linear mixed-effects model (LMM) in R using the *lmerTest* package (Kuznetsova et al., 2017) to test the fixed effects of group (young and older) and periods (different trial periods in the experiment; e.g., early learning, middle learning, and late learning periods) or time delay periods and random effect of subjects on the amount of explicit and implicit learning. To examine the potential difference in the temporal retention of the learning between the two groups, we fitted the absolute angular decay and the relative decay percentage (see above) using an LMM with fixed effects of group and learning type, a random effect of subjects, and actual elapsed time as a covariate. By considering the actual elapsed time as a continuous covariate, we were able to compare the retention between the two age groups across a continuous delay range (i.e., the short-term temporal stability of learning). The LMM models were estimated using the restricted maximum likelihood method and the significance was obtained using Kenward–Roger and Satterhwaite approximations with the *pbkrtest* package (Halekoh and Højsgaard, 2014). If significance was identified, *post hoc* tests were performed using the *emmeans* package (Lenth et al., 2020) and adjusted for multiple comparisons using Bonferroni–Holm corrections. Effect size (*d*) was calculated using Cohen’s *d* (Cohen, 1988) measurement (for LMM analysis, generalized effect size was computed following similar procedures of Cohen’s *d* measurement using the *eff_size* function in the *emmeans* package). For all tests, the significance level was set to 0.05. In all cases, group data are presented as the mean ± SEM. Please see the statistical table for a summary of the data structure, analysis, and confidence intervals (CIs; Table 1).

**Table 1:** Statistics table for “normal aging affects the short-term temporal stability of implicit, but not explicit, motor learning following visuomotor adaptation”

Line #	Data structure	Type of test	95% CI
280	Normal	Two-tailed unpaired *t* test	Baseline overall reaching angles: Young: [−0.026°, 0.93°] Older: [−0.14°, 1.08°]
281	Normal	Two-tailed one-sample *t* test	
284	Normal	Two-tailed unpaired *t* test	Baseline explicit learning: Young: [−0.13°, 0.068°]; older: [−0.24°, 0.18°] Baseline implicit learning: Young: [−0.014°, 0.98°]; older: [−0.17°, 1.17°]
285	Normal	Two-tailed one-sample *t* test	
288	Normal	Linear mixed-effects model: Explicit and implicit learning during baseline ∼ group × learning type + (1|subject)	
312	Normal	Linear mixed-effects model Overall learning ∼ group × training period + (1|subject)	Overall learning during training: Young: early, [7.96°, 17.24°]; middle, [36.92°, 48.19°]; late, [43.29°, 48.99°] Older: early, [9.22°, 16.75°]; middle, [36.95°, 48.12°]; late, [40.35°, 48.88°]
338	Normal	Linear mixed-effects model Explicit and implicit learning during training ∼ group × training period × learning type + (1|subject)	Explicit learning: Young: early, [2.94°, 13.12°]; middle, [14.90°, 26.19°]; late, [18.23°, 29.38°] Older: early, [0.95°, 8.22°]; middle, [11.58°, 21.23°]; late, [12.25°, 27.88°] Implicit learning: Young: early [1.29°, 8.50°]; middle [17.038°, 26.98°]; late [16.75°, 27.92°] Older: early, [4.84°, 11.91°]; middle; [20.11°, 28.19°]; late, [18.14°, 30.96°]
364	Normal	Two-tailed unpaired *t* test	Selection time during baseline: Young: [0.66 s, 1.00 s] Older: [1.02 s, 1.89 s]
371	Normal	Linear mixed-effects model Selection time during training ∼ group × training period + (1|subject)	Selection time during training Young: early, [3.17 s, 6.18 s]; middle, [1.80 s, 2.66 s]; late, [1.60 s, 2.27 s] Older: early, [6.11s, 11.86s]; middle, [4.36s, 9.23s]; late, [2.92s, 6.58s]
432	Normal	Two-tailed paired *t* test	Overall learning during retraining vs 0 s: Young: retraining, [44.14°, 45.68°]; 0 s, [44.21°, 45.72°]
434	Normal	Two-tailed paired *t* test	Overall learning during retraining vs 0 s: Older: retraining, [40.87°, 43.76°]; 0 s, [39.80°, 43.11°]
436	Normal	Linear mixed-effects model Absolute temporal decay of the overall adaptation ∼ group × actual elapsed time + (1|subject)	Absolute temporal decay of the overall learning: Young: 0 s, [−0.77°, 0.92°]; 5 s, [−2.96°, −0.85°]; 10 s, [−2.45°, 0.0192°]; 20 s, [−3.67°, −1.04°]; 30 s, [−4.11°, −0.40°]; 50 s, [−5.04°, −1.76°]; 90 s, [−5.49°, −2.12°] Older: 0 s, [−1.65°, 0.99°]; 5 s, [−2.70°, −0.86°]; 10 s, [−3.30°, −0.66°]; 20 s, [−5.45°, −1.59°]; 30 s, [−5.12°, −1.90°]; 50 s, [−7.06°, −2.37°]; 90 s, [−11.07°, −5.82°]
475	Normal	Linear mixed-effects model Absolute temporal decay of the explicit and implicit learning ∼ group × actual elapsed time × learning type + (1|subject)	Absolute temporal decay of the explicit learning: Young:& 0 s, [−0.89°, 0.83°]; 5 s, [−0.61°, 1.02°]; 10 s, [−0.78°, 1.06°]; 20 s, [−1.07°, 0.41°]; 30 s, [−0.78°, 0.84°]; 50 s, [−0.30°, 0.76°]; 90 s, [−0.99°, 0.94°] Older: 0 s, [−0.63°, 0.16°]; 5 s, [−1.02°, 0.18°]; 10 s, [−0.63°, 0.22°]; 20 s, [−1.08°, 0.51°]; 30 s, [−0.58°, 0.57°]; 50 s, [−0.96°, 0.30°]; 90 s, [−0.84°, 0.58°] Absolute temporal decay of the implicit learning: Young: 0 s, [−1.23°, 1.14°]; 5 s, [−2.92°, −0.13°]; 10 s, [−2.64°, 0.63°]; 20 s, [−4.61°, −0.75°]; 30 s, [−3.74°, −0.25°]; 50 s, [−4.63°, −1.85°]; 90 s, [−5.70°, −1.44°] Older: 0 s, [−1.48°, 1.14°]; 5 s, [−1.97°, 0.36°]; 10 s, [−2.78°, 0.32°]; 20 s, [−4.84°, −1.48°]; 30 s, [−5.81°, −1.66°]; 50 s, [−5.77°, −1.61°]; 90 s, [−9.83°, −6.12°]
504	Normal	Linear mixed-effects model Relative temporal decay of the explicit and implicit learning ∼ group × actual elapsed time × learning type + (1|subject)	Relative temporal decay of the explicit learning: Young: 0 s, [−2.98%, 4.02%]; 5 s, [−1.69%, 5.54%]; 10 s, [−3.13%, 3.67%]; 20 s, [−2.67%, 2.63%]; 30 s, [−3.86%, 2.81%]; 50 s, [−1.50%, 4.32%]; 90 s, [−3.40%, 3.79%] Older: 0 s, [−3.71%, 3.38%]; 5 s, [−9.34%, 2.55%]; 10 s, [−4.21%, 1.75%]; 20 s, [−6.26%, 4.34%]; 30 s, [−4.66, 9.25%]; 50 s, [−9.89%, 4.22%]; 90 s, [−4.82%, 5.19%] Relative temporal decay of the implicit learning: Young: 0 s, [−8.37%, 5.45%]; 5 s, [−16.04%, −1.63%]; 10 s, [−14.04%, 0.24%]; 20 s, [−19.36%, −5.20%]; 30 s, [−19.03, −1.50%]; 50 s, [−21.68%, −7.82%]; 90 s, [−23.88%, −6.41%] Older: 0 s, [−12.49%, 3.40%]; 5 s, [−6.78%, 3.03%]; 10 s, [−14.86%, −0.92%]; 20 s, [−19.27%, −1.83%]; 30 s, [−19.10%, −3.03%]; 50 s, [−28.13%, −9.73%]; 90 s, [−37.36%, −15.57%]
518	Normal	Two-tailed paired *t* test	Selection time during retraining vs. end of training Young: retraining, [1.34 s, 1.93 s]; end of training [1.60 s, 2.27 s]
519	Normal	Two-tailed paired *t* test	Selection time during retraining vs end of training Older: retraining, [2.30 s, 5.19 s]; end of training, [2.92 s, 6.58 s]
521	Normal	Linear mixed-effects model Selection time during adaptation decay ∼ group × delay period + (1|subject)	Selection time during adaptation decay: Young: 0 s, [1.68 s, 2.65 s]; 5 s, [2.14 s, 3.05 s]; 10 s, [2.28 s, 3.31 s]; 20 s, [2.40 s, 3.33 s]; 30 s, [2.48 s, 4.06 s]; 50 s [2.66 s, 3.98 s]; 90 s [2.68 s, 3.77 s] Older: 0 s, [2.40 s, 4.87 s]; 5 s [3.63 s, 6.53 s]; 10 s [4.04 s, 6.53 s]; 20 s [4.73 s, 8.25 s]; 30 s [4.82 s, 8.43 s]; 50 s [4.31 s, 7.19 s]; 90 s [4.74 s, 8.26 s]
535	Normal	Two-tailed unpaired *t* test	Selection time during retraining Young: [1.34 s, 1.93 s] Older: [2.30 s, 5.19 s]
541	Normal	Linear mixed-effects model Change of selection time during adaptation decay ∼ group × delay period + (1|subject)	Change of selection time during adaptation decay: Young: 0 s, [0.27 s, 0.80 s]; 5 s [0.68 s, 1.15 s]; 10 s, [0.83 s, 1.52 s]; 20 s, [0.93 s, 1.53 s]; 30 s, [1.00 s, 2.32 s]; 50 s, [1.23 s, 2.10 s]; 90 s, [1.25 s, 2.02 s] Older: 0 s, [−0.59 s, 1.17 s]; 5 s, [−0.05 s, 2.87 s]; 10 s, [0.48 s, 2.59 s]; 20 s, [1.96 s, 4.41 s]; 30 s, [1.97 s, 4.47 s]; 50 s, [1.60 s, 3.45 s]; 90 s, [1.97 s, 4.59 s]

## Results

### Baseline performance

All subjects practiced selecting the aiming direction and reaching to the targets during the baseline block with 20% blank no-visual-feedback trials (a total of eight blank movements). During the baseline trials, all participants in both groups moved the cursor directly to the target, and the reach angles were not significantly different from each other (young: 0.45° ± 0.23°; older: 0.47° ± 0.29°; two-tailed unpaired *t* test: *p *=* *0.97, *d* = 0.012) and not significantly different from 0° (two-tailed one-sample *t* test; young group: *p *=* *0.16, *d* = 0.37; older group: *p *=* *0.12, *d* = 0.44). Participants demonstrated no explicit aiming or implicit learning during this block, as expected (explicit: young, −0.029° ± 0.046°; older, −0.031° ± 0.10°; two-tailed one-sample *t* test; young group: *p *=* *0.53, *d* = 0.14; older group: *p *=* *0.75, *d* = 0.073; implicit: young, 0.48° ± 0.24°; older, 0.50° ± 0.32°; two-tailed one-sample *t* test; young group: *p *=* *0.06, *d* = 0.45; older group: *p *=* *0.13, *d* = 0.36). Additionally, there were no significant differences between the explicit and implicit components of adaptation in both groups (an analysis using LMM with fixed effects of group and learning type and random effect of subjects: group: *F*_(1,74)_ = 0.0012, *p *=* *0.97; learning type: *F*_(1,74)_ = 0.35, *p *=* *0.12; interaction between the two fixed effects: *F*_(1,74)_ = 0.0021, *p *=* *0.96).

### Adaptation to the visual feedback perturbation

**Figure 2.**
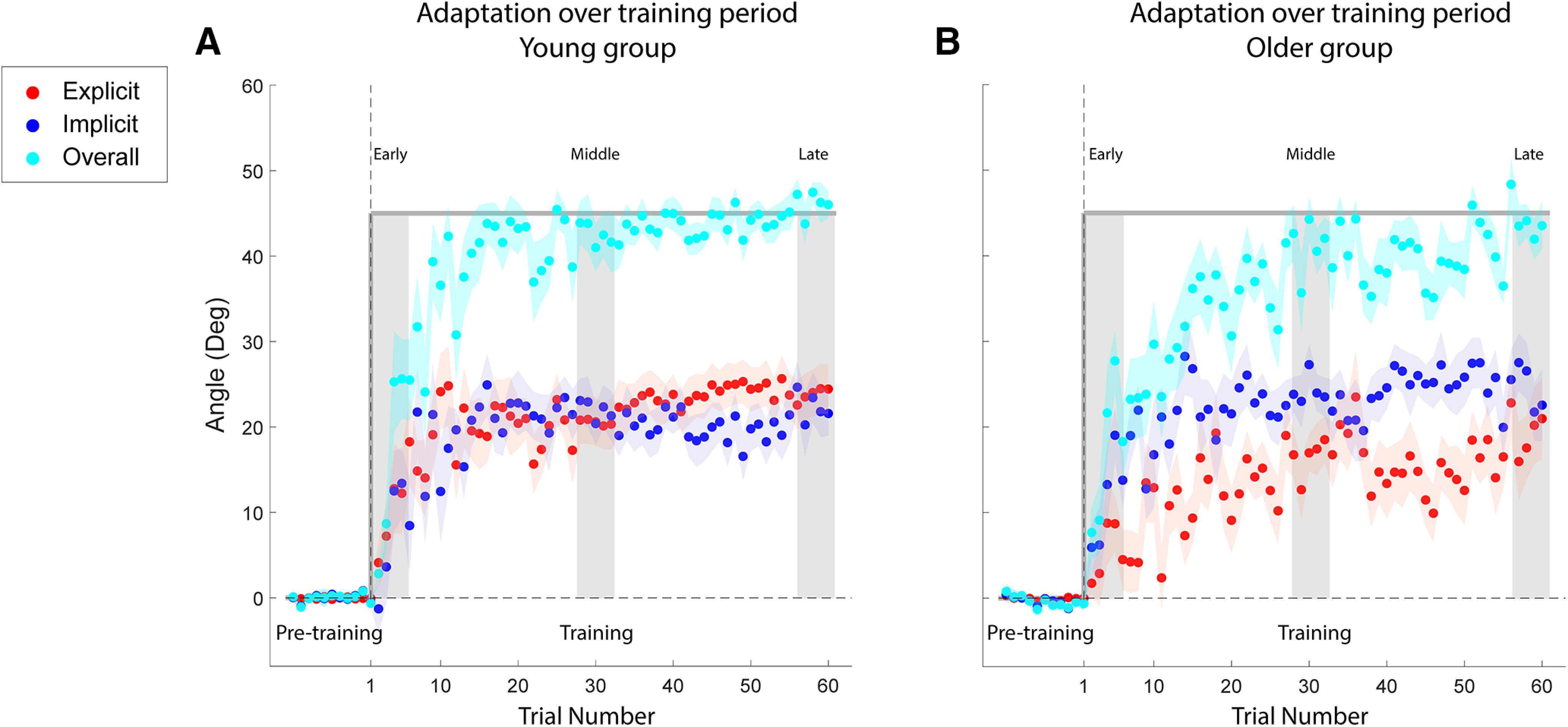
Adaptation curves over the training period. ***A***, ***B***, The learning curves for young (***A***) and older (***B***) subjects during training. The *x*-axis represents the progression of trials, divided into pretraining (baseline, without the visuomotor rotation perturbation) and training (which was composed of 60 trials). The dark gray trace represents the magnitude of the rotation of visual feedback, and light gray vertical bars are the three periods during training (early: the first five trials; middle: the middle five trials; and late: the last five trials). Overall and implicit adaptation, and explicit aiming levels were measured in units of degrees, through the methods depicted by Figure 1*E*. The light blue symbols represent the mean overall adaptation on each trial, while red and dark blue symbols represent the mean explicit and implicit adaptation levels on each trial, respectively. Background shading represents the SEM.

After the baseline period, a 45° VMR (CW or CCW) was introduced for 60 trials (Fig. 2*A*,*B*). After the perturbation, the older subjects’ overall learning (Fig. 2*B*, light blue trace) generally progressed in a pattern similar to that of the young subjects (Fig. 2*A*, light blue trace). We first evaluated the mean adaptation over the training period for both groups. Throughout the entire training period, the subjects in the young group had a significantly higher explicit learning level (young group: 17.73° ± 3.40°; older group: 11.56° ± 3.6°; two-tailed unpaired *t* test, *p *> 0.022, *d* = 0.76) and a similar implicit learning level compared with the subjects in the older group (young group: 16.35° ± 3.43°; older group: 18.65° ± 3.64°; two-tailed unpaired *t* test, *p *=* *0.26, *d* = 0.37), leading to significantly higher overall adaptation for the young group (young group: 34.07° ± 4.55°; older group: 30.20° ± 4.68°; two-tailed unpaired *t* test, *p *>* *0.023, *d* = 0.75). To better investigate the progression of the learning and the potential difference in adaptation, we compared the learning between the two groups at three periods during training (early, middle training, and late training; see Materials and Methods). Over the entire training block, both groups demonstrated an increase in overall learning. During the three training periods, older subjects adapted at an overall rate similar to that in young subjects (early training: young group, 12.60° ± 2.22°; older group, 13.04° ± 1.79°; middle training: young group, 42.56° ± 2.69°; older group, 42.59° ± 2.65°; late training: young group, 46.14° ± 1.36°; older group, 44.67° ± 2.01°). An LMM analysis with a random effect of subjects was used to examine two fixed effects of group (young and older) and training period (early, middle, and late training) on overall learning. This showed that training period had a significant effect on overall learning (*F*_(2,74)_ = 185.61, *p *<* *0.001), but there was not a significant effect of group (*F*_(1,37)_ = 0.032, *p *=* *0.85) or an interaction between the two fixed effects (*F*_(2,74)_ = 0.144, *p *=* *0.86). *Post hoc* tests showed that subjects in both groups adapted to the VMR perturbation rapidly and their overall learning during the middle training period was significantly greater than the learning during the early training period (young group: *p *<* *0.001, *d* = 3.62; older group, *p *<* *0.001, *d* = 3.57). All subjects almost reached their asymptotic performance during the middle training period, and their learning did not increase significantly between the middle and late training periods (young group, *p *=* *0.95, *d* = 0.43; older group, *p *=* *0.52, *d* = 0.25).

**Figure 3.**
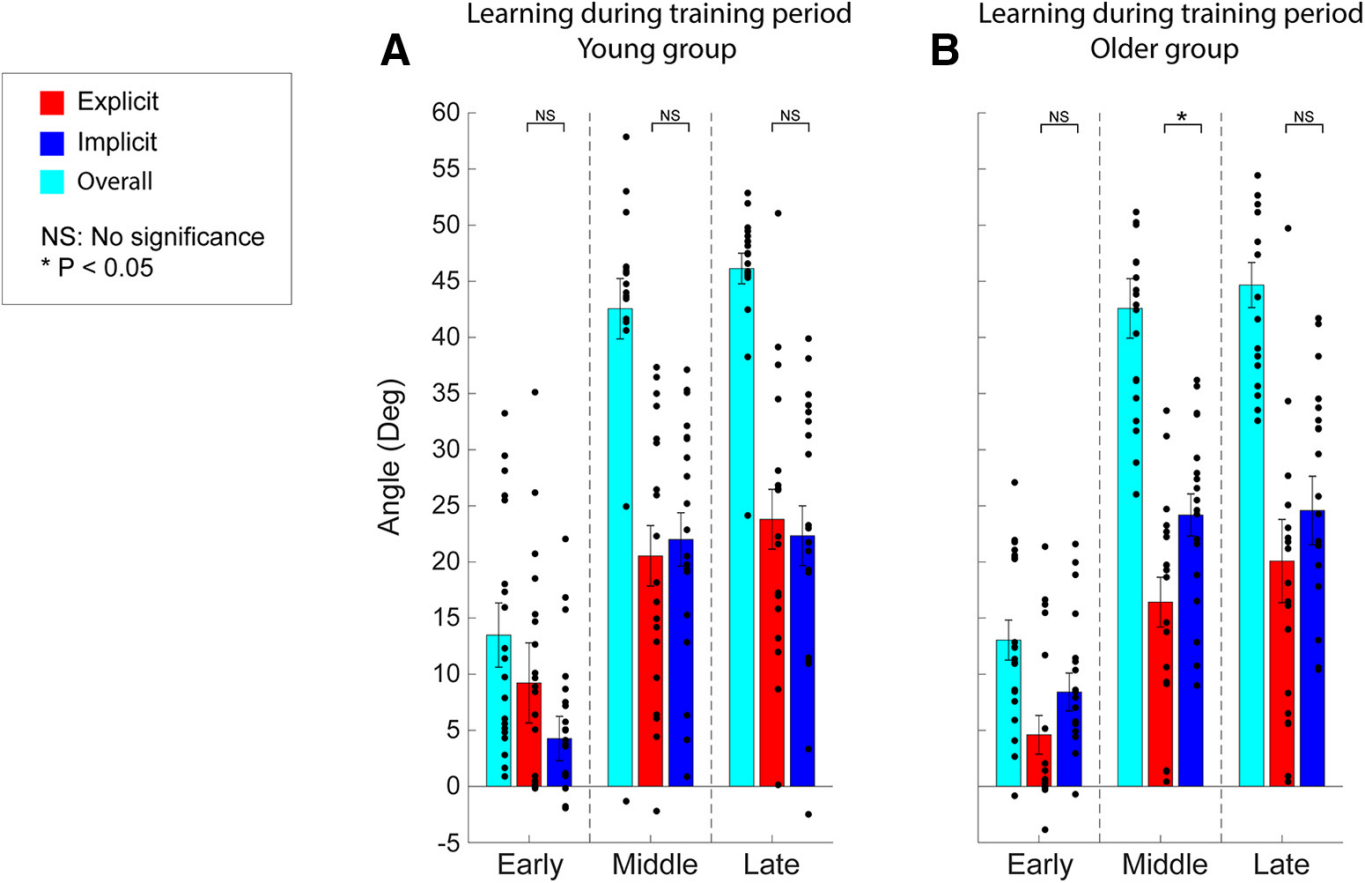
Overall adaptation, and levels of explicit and implicit learning during training. ***A***, ***B***, Histograms of the average amount of explicit, implicit, and overall adaptation that occurred for young (***A***) and older (***B***) subject groups during the early, middle, and late training periods. Overall, explicit, and implicit adaptation were measured in degrees, as described in Figure 1*E*. The light blue symbols represent the overall adaptation, while red and dark blue symbols represent the explicit and implicit adaptation levels, respectively. The individual data for each subject are represented by the black filled circles next to the respective histogram. Vertical error bars represent the SEM.

Despite similarities in the overall adaptation, the subject groups varied in their patterns of explicit and implicit adaptation (Fig. 2*A*, red trace, *B*, dark blue trace). During early training, the young subjects (Fig. 3*A*) demonstrated a greater increase in their explicit aiming than in their implicit learning (explicit, 8.03° ± 2.43°; implicit, 4.89° ± 1.72°). The explicit and implicit learning curves for the young subjects both reached an asymptote in the middle of the training block at similar levels (explicit, 20.55° ± 2.70°; implicit, 22.01° ± 2.38°), and both explicit and implicit learning components remained at similar levels at the end of the training (explicit, 23.80° ± 2.66°; implicit, 22.34° ± 2.67°). This pattern differed from that in the older subjects (Fig. 3*B*), in that during early training the explicit and implicit learning components had the opposite relationship compared with young subjects (explicit, 4.58° ± 1.73°; implicit, 8.41° ± 1.69°). In the middle training period, implicit learning remained greater than explicit aiming (explicit, 16.42° ± 2.22°; implicit, 24.19° ± 1.87°). Similar to the young subjects, as training progressed for the older subjects, the two learning components converged to similar levels at the end of the training session (explicit, 20.08° ± 3.71°; implicit, 24.58° ± 3.06°). An LMM with random effects of subjects was used to examine the fixed effects of group, learning type, and training period on the learning levels acquired by subjects in both groups. We found that there was a significant main effect of training period (*F*_(1,220)_ = 51.05, *p *<* *0.001) and an interaction between group and learning type (*F*_(1,220)_ = 4.94, *p *=* *0.028). However, there were no other significant interactions among the three fixed effects (interaction between training period and group: *F*_(2,220)_ = 0.046, *p *=* *0.95; interaction between training period and learning type: *F*_(2,220)_ = 0.78, *p *=* *0.46; interaction among the three fixed effects: *F*_(2,220)_ = 0.010, *p *=* *0.98). *Post hoc* tests showed a significant increase in both explicit and implicit learning components from early training to middle training for both groups (*p *<* *0.0038, d > 1.08 for all cases), with no significant change beyond the middle training period (all cases: *p *>* *0.93, *d* < 0.33). During early and late training, explicit and implicit learning components were not significantly different for both groups (young subjects: early, *p *=* *0.36, *d* = 0.29; late, *p *=* *0.67, *d* = 0.13; older subjects: early, *p *=* *0.28, *d* = 0.35; late, *p *= 0.20, *d* = 0.41). However, during middle training, there was a significant difference between the explicit and implicit components for older subjects (*p *=* *0.035, *d* = 0.71), while the difference was not significant for younger subjects (*p *=* *0.67, *d* = 0.13). The two learning components were also compared across the two age groups in the *post hoc* tests. We found that for all training periods, subjects in the two groups applied similar levels of explicit aiming (three training periods: *p* > 0.24, *d* < 0.38) and implicit learning (all three training periods: *p* > 0.32, *d* < 0.31) to counter the perturbation.

### Selection time of explicit aim direction during training

**Figure 4.**
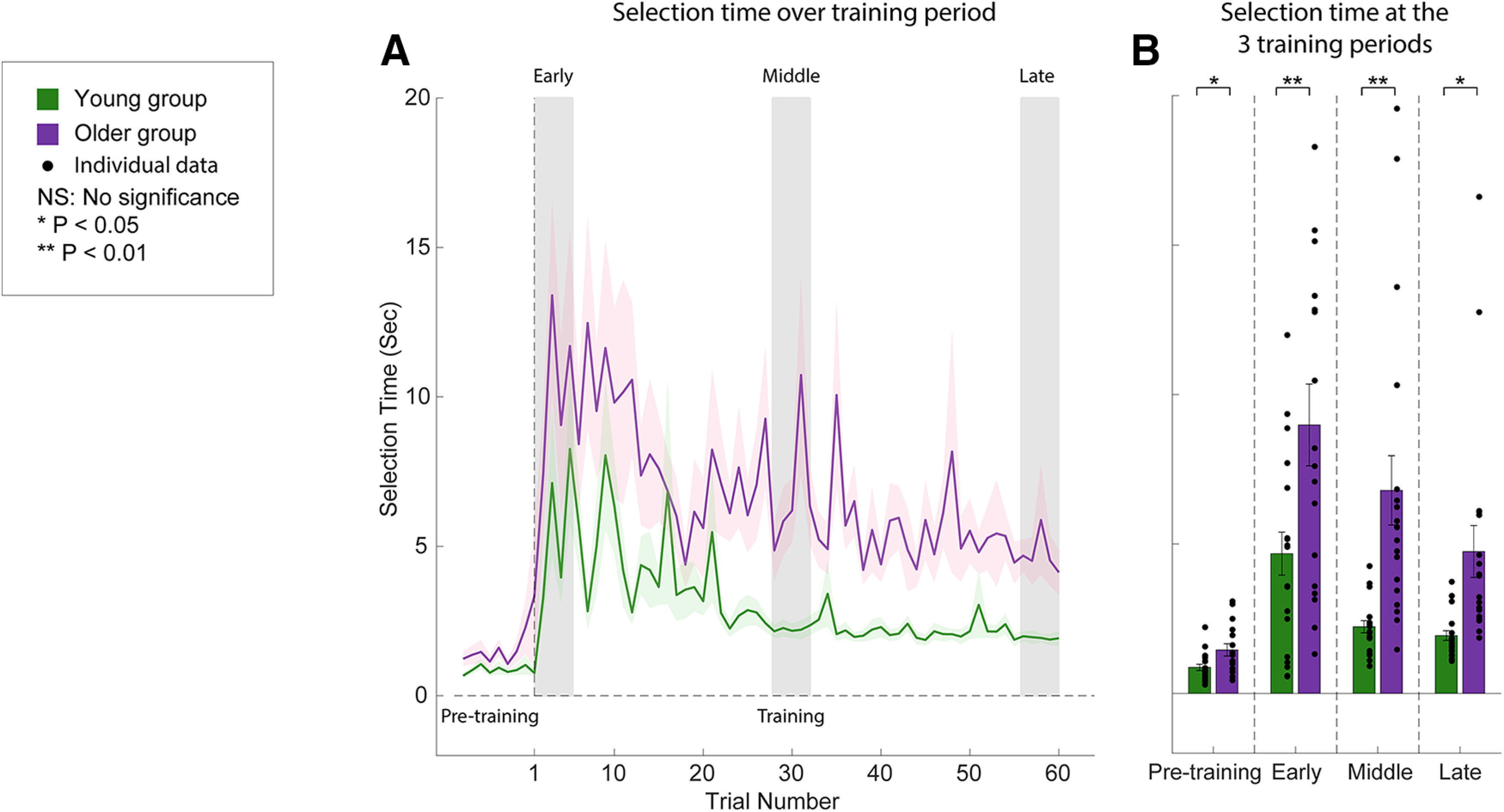
Explicit movement direction selection time over the training period. ***A***, Selection time was plotted as a function of trial number during pretraining (baseline, without the visuomotor rotation perturbation) and training period (which was composed of 60 trials). The young and older subject groups are represented by green and purple traces, respectively. ***B***, Histograms of the selection time at the three training periods (early, middle, and late) for young and older subjects. The individual data for each subject are represented by the black filled circles next to the respective histogram. Vertical error bars represent the SEM.

We also compared the selection time for the direction report (see Materials and Methods) between the two groups during training (Fig. 4*A*,*B*). For the eight baseline trials, we observed that older subjects required significantly more selection time, although the VMR was not applied (young, 0.87 ± 0.10 s; older, 1.46 ± 0.21 s; two-tailed unpaired *t* test: *p *=* *0.015, *d* = 0.82). This indicates that older subjects initially required a longer time to operate the knob. Interestingly, both groups required a large selection time to choose their aiming directions after the first exposure to the VMR in early training (young, 4.68 ± 0.72 s; older, 9.00 ± 1.37 s). For both groups, the changes in the selection time followed a similar pattern as subjects learned the task, with an initial sharp increase and reaching an asymptote at the end of the training, albeit at different levels (late training: young, 1.94 ± 0.16 s; older, 4.75 ± 0.87 s). An LMM with random effect of subjects was used to test the fixed effects of group and training period (early, middle, and late) on the selection time during training. We found that both effects were significant (main effect of group: *F*_(1,74)_ = 10.69, *p *<* *0.001; main effect of training period: *F*_(2,37)_ = 22.42, *p *<* *0.001) with no interaction between them (*F*_(2,74)_ = 0.76, *p *=* *0.47). This was also the case when the initial baseline differences were taken into account. *Post hoc* tests showed that the selection time for subjects in both groups significantly decreased at the end of training compared with early training (young group: *p *=* *0.038, *d* = 0.81; older group, *p *=* *0.001, *d* = 1.25). For all training periods, older subjects required a significantly longer selection time than young subjects (Fig. 4*B*; training periods: *p* < 0.022, *d* > 0.83). Thus, at the end of training, although subjects in the two groups fully learned the VMR and adopted similar levels of explicit strategy to compensate for the perturbation (Figs. 2, 3), older subjects still required a significantly longer selection than young subjects.

**Figure 5.**
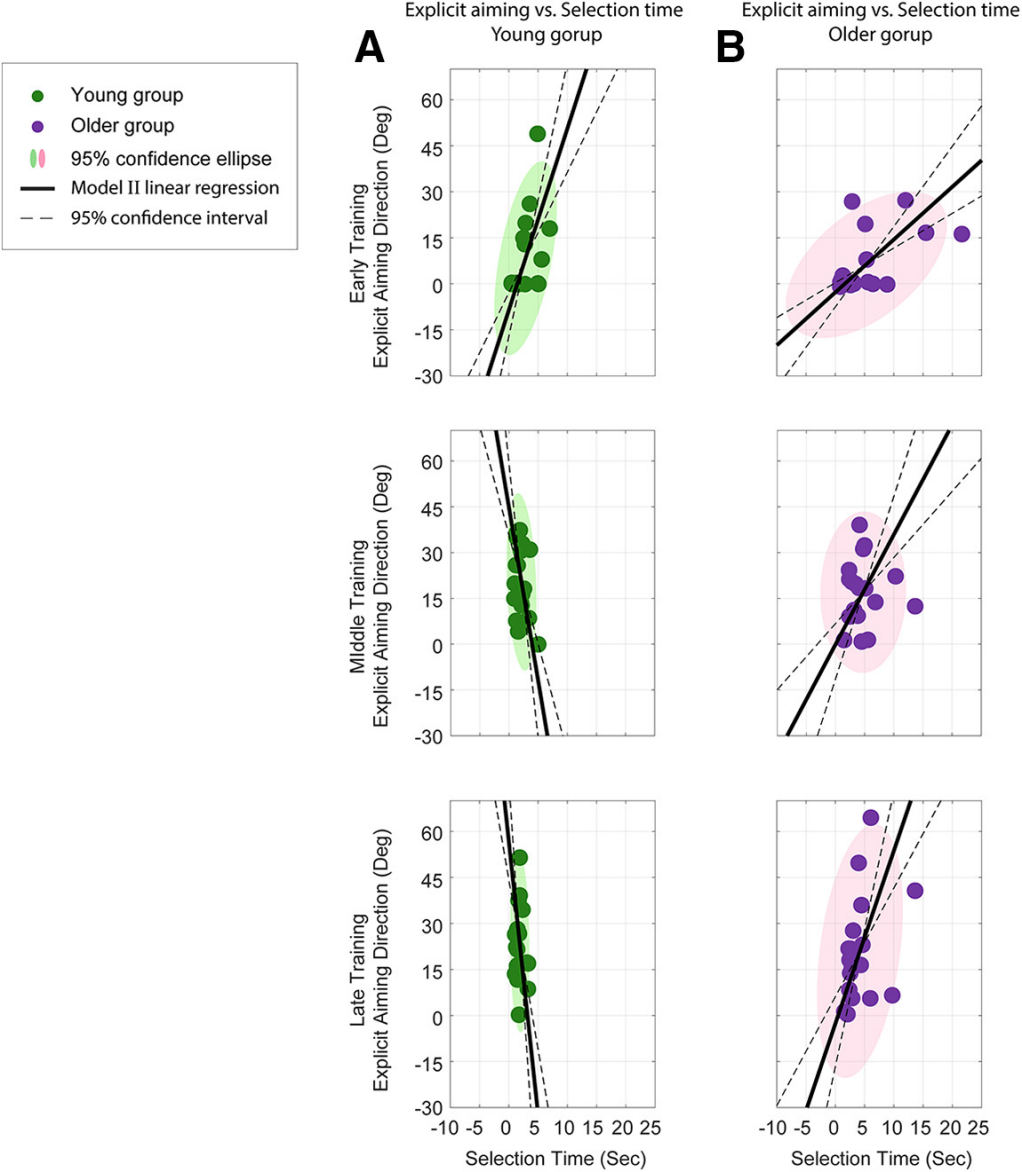
Relationship between explicit aiming direction and selection time during adaptation. ***A***, ***B***, Explicit aiming directions during the three training periods (early, middle, and late) as a function of selection time for young (***A***) and older (***B***) subjects. The young and older subject groups are represented by green and purple symbols, respectively. Each filled circle represents the mean explicit aiming direction and the corresponding selection time during each training period for each subject. The shaded ellipses are the 95% confidence ellipses of the data. The black solid lines represent model type II linear regression fits to the data, and the black dashed lines represented the 95% confidence intervals for the regression.

To further examine the relationship between explicit aiming and selection time, we plotted the former as a function of the latter in Figure 5. As subjects began to develop an explicit aiming strategy during early training (Fig. 5, top row), the major axes of the ellipses were oriented toward a positive slope for both age groups, indicating that the explicit aiming directions increased as subjects used a longer selection time. After further adaptation during the middle and late training periods (Fig. 5, middle and bottom rows, respectively), the confidence ellipses for both groups rotated counterclockwise and their major axes oriented toward similar directions along the *y*-axis (90°). This change in orientation is reflected in Figures 2 and 4; over the training period, subjects in the respective groups selected similar aiming directions (Fig. 2, asymptotes, red traces) within a shorter time (Fig. 4, asymptotes, purple and green traces). However, as noted above, older subjects still required a longer selection time than the young subjects, which is demonstrated by the wider minor axes of the corresponding confidence ellipses. To quantify the relationship between explicit aiming and selection time, we applied a model type II linear regression (using the *lmodel2* package in R; Legendre, 2018) on the two variables (because both explicit aiming and selection time are noisy measurements, we used model II linear regression instead of model I). The black solid lines in Figure 5 are the model II regression fit, and the black dashed lines are the 95% CIs for the regression. During the early training period, the slopes for the linear regressions were significant for both groups (young subjects: 5.92; 95% CI, [3.93, 8.92]; *p *<* *0.02; older subjects: 1.72; 95% CI, [1.13, 2.62]; *p *<* *0.05). During the middle and late training periods, the slopes of the linear regression became steeper for both groups showing that subjects were able to use a shorter time to select their aiming directions (middle training: young subjects, −11.32; 95% CI, [−18.02, −7.11]; *p *=* *0.12; older subjects, 3.60; 95% CI, [2.16, 5.97]; *p *=* *0.42; late training: young subjects, −17.81; 95% CI, [−28.63, −11.08]; *p *=* *0.40; older subjects, 5.64; 95% CI, [3.55, 8.97]; *p *=* *0.11). Furthermore, the slopes of the regression were not significant for both groups during middle and late training, indicating that across subjects the explicit aiming strategy used to compensate for the visuomotor rotation did not significantly change with selection time.

### Temporal decay of implicit and explicit learning

**Figure 6.**
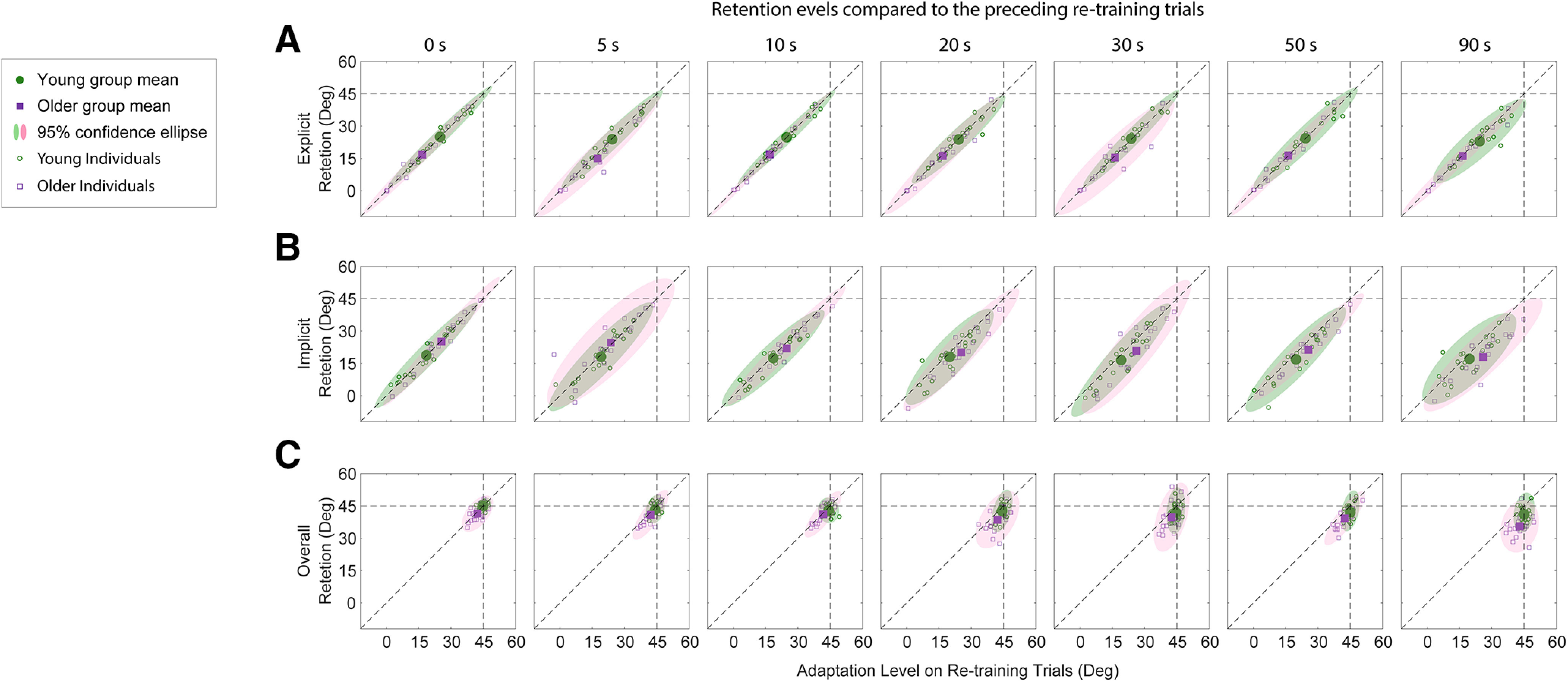
Retention of learning with respect to previous retraining trials. ***A–C***, The amount of learning (aiming and reaching angle) on the probe trial is plotted against the amount of learning on the previous retraining trials for explicit aiming (***A***), implicit learning (***B***), and overall learning (***C***) for the seven different delays (each respective column). The individual data for each subject are represented by the green circles (young subjects) and purple squares (older subjects). The group mean in each panel is depicted by the larger filled symbols for each respective group, and the shaded ellipses represent the 95% confidence intervals. Horizontal and vertical dashed lines show the maximum VMR perturbation (45°), and the diagonal dashed line is the unity line.

We first assessed the retention of explicit aiming, and implicit and overall learning by comparing the aiming angles and reaching angles on the nonvisual feedback probe trials to the average aiming and reaching angles over the last three retraining trials (see Materials and Methods). We plotted the former as a function of the latter for each individual probe trial in Figure 6. For the explicit learning (Fig. 6*A*), the majority of the data points for both groups were on the diagonal unity lines for the different delay periods, indicating no obvious decay over time (note the mean values in each panel of Fig. 6 are on the unity lines). For the implicit learning and overall learning (Fig. 6*B*,*C*), most data points started to fall below the unity line as the delay period increased. Also, the data points for the older group (Fig. 6*B*,*C*, purple squares) deviated further from the diagonal unity lines than those for the young group (Fig. 6*B*,*C*, green circles), suggesting greater decay with time (Fig. 6*B*,*C*, the mean values are below the unity line, with the data for the older group below the young).

**Figure 7.**
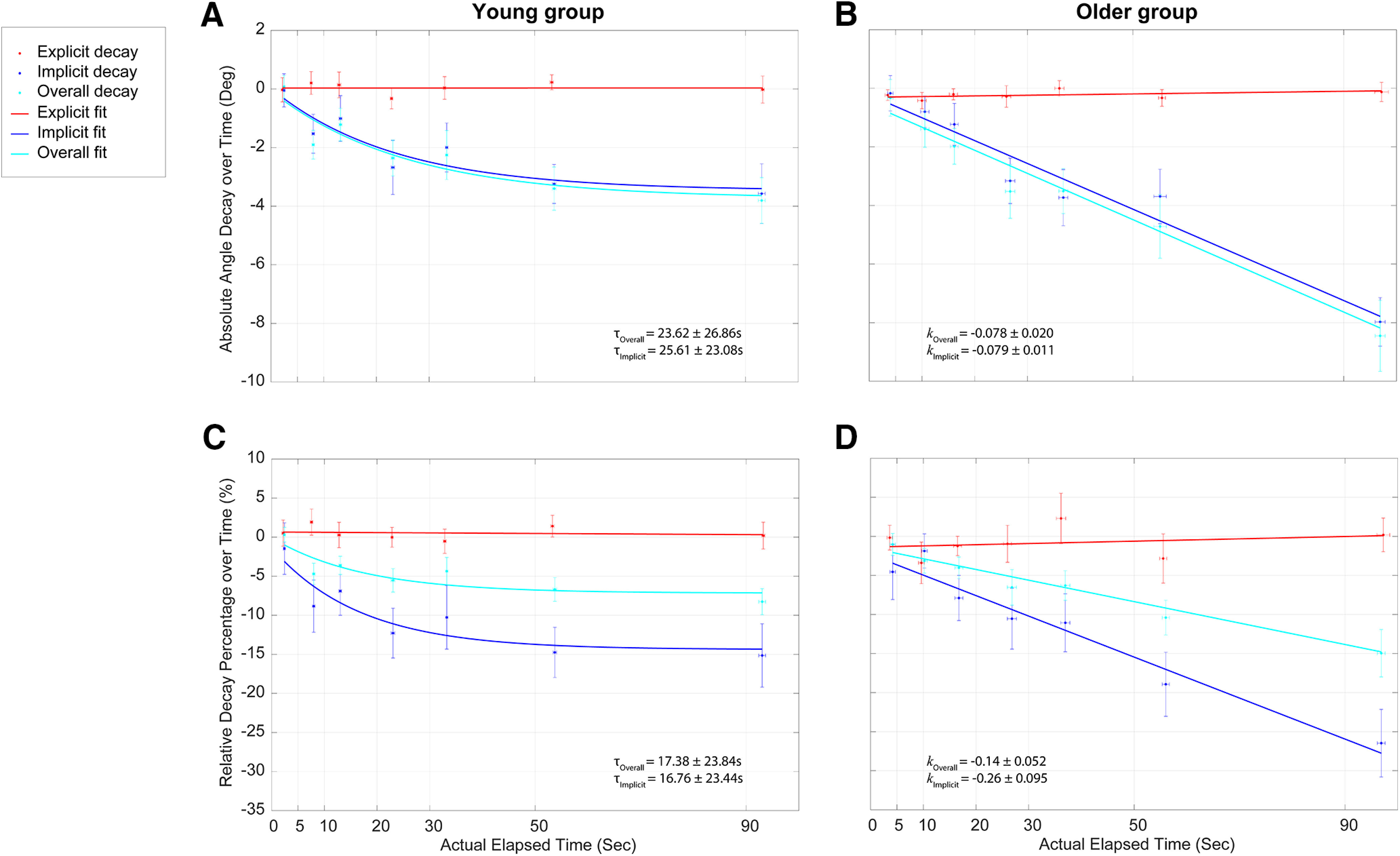
Temporal decay curves. ***A–D***, The decay of implicit, explicit and overall learning as a function of the actual elapsed time is shown for young (***A***, ***C***) and older (***B***, ***D***) subjects. The actual elapsed time is the time interval from the end of the previous trial to the time when the selection was made for the explicit learning component, and the time interval from the end of previous trial to the movement onset for the implicit learning component. Note that the actual elapsed time was always slightly longer for the implicit learning component. The absolute retention (angular deviation) and relative retention (percentage of the adaptation at the end of preceding retraining trials) are shown on the top and bottom rows, respectively. The mean value for each respective measure (overall learning: light blue symbols; explicit aiming: red symbols; implicit learning: dark blue symbols) on the probe trial is plotted for the actual elapsed time for each respective delay period (0, 5, 10, 20, 30, 50, and 90 s). Each panel includes the best fit function (***A***, ***C***, exponential for young subjects; ***B***, ***D***, linear for older subjects) for the decay of overall adaptation and implicit learning. Explicit learning for each group was best described by a linear function. Vertical error bars represent the SEM in the respective measures of decay, and horizontal error bars represent the SEM in the actual elapsed time.

To better observe the difference between groups and to quantify the retention of learning, absolute angular decay (Fig. 7*A*) and relative learning decay (Fig. 7*B*) for the probe trials compared with the end of the preceding retraining trials were examined (see Materials and Methods). For angular decay (Fig. 7*A*), at the delay of 0 s, the amount of compensation for the VMR was not significantly different from the amount of overall recalibration during retraining for both groups (young group: retraining, 44.94° ± 0.66°; 0 s, 44.97° ± 0.81°; two-tailed paired *t* test, *p *>* *0.94, *d* = 0.0065; older group: retraining, 42.29° ± 0.91°; 0 s, 41.47° ± 1.22°; two-tailed paired *t* test, *p *>* *0.48, *d* = 0.17). To investigate the difference in temporal decay of overall adaptation (reaching angles) between the two groups, we fitted the angular decay data using an LMM with fixed effects of group, random effect of subjects, and actual elapsed time as a covariate. Throughout the delay periods (range, 0–90 s), the temporal decay of the overall adaptation for young subjects (Fig. 6*A*, light blue circles) was consistent with previous studies (Hadjiosif and Smith, 2013; Zhou et al., 2017). There were significant effects of group and delay period on the retention of overall learning (main effect of group: *F*_(1,51.68)_ = 10.55, *p *=* *0.002; main effect of delay period: *F*_(1,215.34)_ = 85.73, *p *=* *0.0001), and the interaction between the two effects was also significant (*F*_(1,215.34)_ = 13.60, *p *=* *0.0003). Thus, the overall temporal retention for both groups decayed significantly as the actual delay period increased (values are actual delay and mean delay period: young group: 0 s, 2.47 ± 0.19 s; 5 s, 8.01 ± 0.23 s; 10 s, 13.19 ± 0.27 s; 20 s, 23.11 ± 0.30 s; 30 s, 33.32 ± 0.24 s; 50 s, 53.71 ± 0.33 s; 90 s, 93.07 ± 0.65 s; older group: 0 s, 4.01 ± 0.52 s; 5 s, 10.55 ± 0.81 s; 10 s, 16.14 ± 0.75 s; 20 s, 26.74 ± 0.88 s; 30 s, 36.81 ± 0.86 s; 50 s, 55.16 ± 0.1.19 s; 90 s, 96.91 ± 0.96 s). The decay of the older group was significantly larger than the decay of the young group over time. We fit the decay data with both the exponential and linear decay models (see “Materials and Methods”) and found that the pattern of temporal decay of overall learning for young subjects was well represented by the single exponential decay model (Fig. 6*A*, light blue line; exponential model: *R*^2^ = 0.54, *p *=* *0.00,011, AIC = 627.13; vs linear model: *R*^2^ = 0.45, *p *=* *0.00,013, AIC = 648.20). The time constant of the exponential fit was 25.61 ± 23.08 s, indicating that only a portion of the overall learning was dependent on the passage of time. In contrast to the young subjects, the temporal decay of overall learning for the older group was best described by the linear model (Fig. 6*B*, light blue line; exponential model: *R*^2^ = 0.42, *p *< 0.0001, AIC = 650.39; vs linear model: *R*^2^ = 0.46, *p *< 0.0001, AIC = 641.56). The slope of the linear model *k* was −0.079 ± 0.011. Thus, the overall learning for the two age groups decayed in a significantly different way. The differences in decay rate between the young and older subject groups suggest that there would continue to be significant age-related differences in retention at longer delay periods, since the retention in younger subjects had reached asymptote.

The decay in explicit aiming and implicit learning during the decay process was also assessed. The young subjects demonstrated a near complete retention of explicit aiming across the delay periods (Fig. 7*A*, red filled circles). This trend resembled the same results for the older subjects (Fig. 7*B*, red circles). We fitted the explicit aiming decay for both groups with the linear model and found that the linear model was not significantly different from a constant model (young group: *p *=* *0.99, ŋ^2^ = 0.0001; older group: *p *=* *0.52, ŋ^2^ = 0.0033). That is, there was near constant retention of the explicit aiming direction across the actual elapsed time. However, the retention and decay pattern for implicit learning was very different between the two groups (Fig. 7*A*,*B*, dark blue filled circles). For both groups, the decay of implicit learning was very similar to the decay of the overall learning (young group: time constant, 23.62 ± 26.86 s; older group: slope, −0.078 ± 0.020). This was expected, because, as shown above, the explicit aiming component remained fairly constant over the time delay periods. The analysis using an LMM with a random effect of subjects was performed to examine the effects of actual elapsed time, learning type, and group on the explicit and implicit learning decay. Significance was found for all three fixed effects (actual elapsed time: *F*_(1,488.16)_ = 49.35, *p *<* *0.0001; learning type: *F*_(1,484.68)_ = 156.10, *p *=* *0.0018; group: *F*_(1,75.87)_ = 10.52, *p *=* *0.0018) and interaction among the three fixed effects (*F*_(1,484.89)_ = 9.62, *p *=* *0.002). *Post hoc* tests showed that for both groups, implicit learning decay was significantly larger than the explicit learning decay over time (young group: *t*_ratio(484)_ = 3.02, *p *=* *0.0026; older group: *t*_ratio(486)_ = 7.19, *p *<* *0.0001). However, there was no significant difference in the temporal angular decay of explicit aiming between the two groups (*t*_ratio(486)_ = 0.18, *p *>* *0.85) and temporal decay of the implicit learning for the older group was significantly larger than the decay of the young group (*t*_ratio(484)_ = −4.17, *p *<* *0.0001).

**Figure 8.**
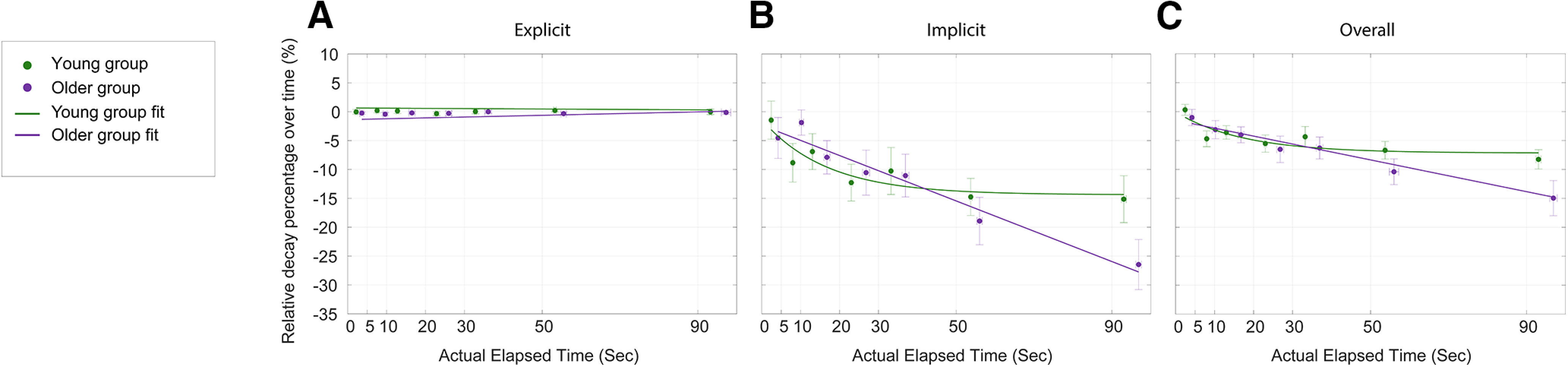
Comparison of the relative retention between the two age groups. ***A–C***, The relative decay percentage for explicit (***A***), implicit (***B***), and overall (***C***) learning (compared with the end of preceding retraining trials). The mean decay percentage for the two groups (young group: green symbols; older group: purple symbols) on the probe trial is plotted for the actual elapsed time for each respective delay period (0, 5, 10, 20, 30, 50, and 90 s), together with the best fit for each dataset (the solid lines). Vertical error bars represent SEM in the respective measures of decay, and horizontal error bars represent the SEM in the actual elapsed time.

To better understand the temporal decay for the two learning components, we also measured the relative retention compared with the end of the preceding retraining trials (Fig. 7*C*,*D*). The relative retention of the overall learning and explicit learning for both groups follows the same trends as the absolute angular retention shown in Figure 7, *A* and *B*. (Fig. 8*A–C* shows a direct comparison of the relative retention of explicit, implicit, and overall learning between the two groups, respectively.) Both groups had similar relative retention of the explicit learning, and the relative overall learning decay for the older group was significantly larger than that for the young group. For actual elapsed time up to 100 s, the average decay of the overall learning for the older group (15%) was about twice the decay for the young group (8%). One interesting result we observed was that the relative decay of the implicit learning for both groups was about twice that of the relative decay of their overall learning. This difference is because of dividing the absolute angular decay by the implicit learning levels at the end of the retraining trials, which accounted for ∼50% of the overall learning (i.e., both explicit and implicit learning contributed ∼50% to the overall learning for both groups at the end of training; Figs. 2, 3). Thus, the relative temporal decay of the implicit learning was actually greater than the relative decay of the overall learning for both groups because of the stability of the explicit aiming (young group: time constant for the implicit decay, 16.76 ± 23.44 s; time constant for the overall decay, 17.38 ± 23.84 s; slope for the implicit decay, −0.26 ± 0.095; slope for the overall decay, −0.14 ± 0.052). The same LMM with random effect of subjects was performed to examine the effects of actual elapsed time, learning type, and age group on the relative temporal decay of the explicit and implicit learning. Significance was found for the interaction among the three fixed effects (*F*_(1,484.89)_ = 4.80, *p *=* *0.029). *Post hoc* tests revealed the same results that relative retention of the explicit learning was not significantly different between the two groups (*t*_ratio(486)_ = 0.36, *p *>* *0.71) and the relative temporal decay of the implicit learning for the older group was significantly larger than the decay of the young group (*t*_ratio(484)_ = −2.69, *p *=* *0.0074).

### Selection time during adaptation decay

**Figure 9.**
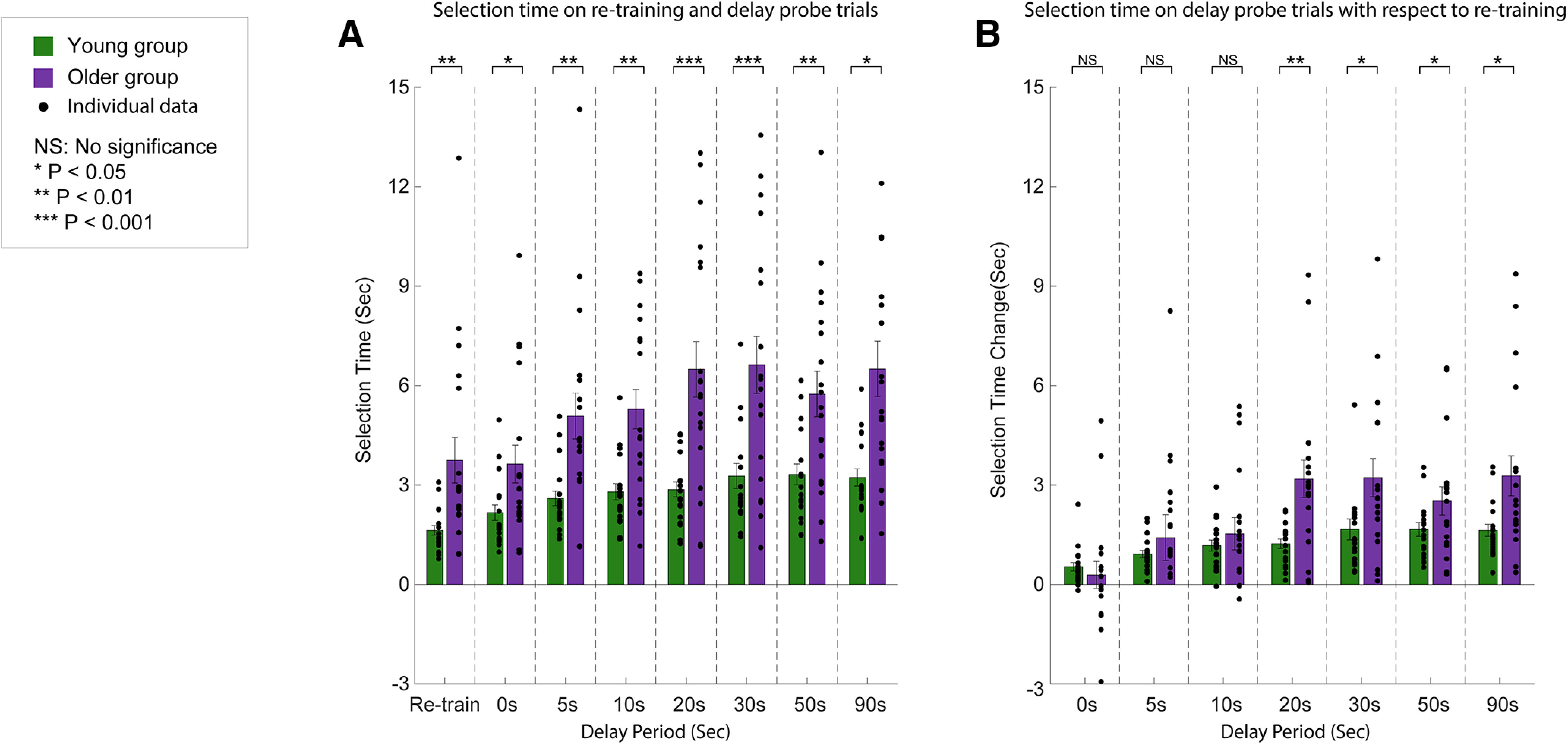
Selection time during the decay period. ***A***, Overall selection time for retraining trials and each delay period is shown for the young (green symbols) and older subjects (purple symbols). ***B***, The change in selection time for each delay period is shown, normalized by the selection time on the preceding retraining trials. The individual data for each subject are represented by the black filled circles next to the respective histogram. Vertical error bars represent the SEM.

We also compared the explicit aiming selection time during the testing phase. We first examined the selection time during retraining (the average of the last three retraining trials) for each delay period for both groups. During retraining, subjects in both groups used a similar selection time compared with their selection time at the end of training, each showing no significant difference (young group: 1.63 ± 0.14 s; two-tailed paired *t* test, *p *=* *0.17, *d* = 0.34; older group: 3.75 ± 0.69 s; two-tailed paired *t* test, *p *>* *0.37, *d* = 0.29). The selection time increased for both subject groups as the delay period increased (Fig. 9*A*). We used an LMM with the random effect of subjects to examine the fixed effects of group and delay period on the selection time and found that the two fixed effects and their interaction were significant (effect of group: *F*_(1,37)_ = 16.18, *p *=* *0.00027; effect of delay period: *F*_(6,222)_ = 13.69, *p *<* *0.00001; interaction: *F*_(6,222)_ = 3.38, *p *=* *0.0033). *Post hoc* tests showed that for all the delay periods >0 s, young subjects did not require significantly greater selection times from their selection time duration at 0 s (5–90 s delay periods: *p *>* *0.05, *d* < 0.48), while older subjects required a significantly longer selection time than the time they required at 0 s (5–90 s delay periods: *p *<* *0.022, *d* > 1.12). For all delay periods (0–90 s), older subjects required a significantly longer selection time than young subjects (all delay periods: *p *<* *0.040, *d* > 1.33).

Despite the significant difference in selection time between the two groups, we were not able to distinguish between whether the older subjects need more time for choosing the explicit aiming direction or they just required more time to operate the knob compared with young subjects. Considering that both groups required significantly different selection times during retraining trials (two-tailed unpaired *t* test, *p *=* *0.0038, *d* = 0.99), we used the selection time in the retraining as the baseline time to operate the knob and examined whether the relative change of the selection time for each delay length remained significantly different. Thus, we took into account differences in the operation time of the knob to determine between group differences specific to forming the explicit aiming strategy. We plotted the change of the selection time for each delay period in Figure 9*A* by subtracting the average selection time in the preceding retraining trials (Fig. 9*B*). The same LMM as above was performed to evaluate the difference in the change of selection time. There was a significant effect of the delay period (*F*_(6,222)_ = 12.01, *p *<* *0.00001) but no significant effect of group (*F*_(1,37)_ = 2.37, *p *=* *0.14). The interaction between the effects of group and delay period was significant (*F*_(6,222)_ = 3.20, *p *<* *0.0051). *Post hoc* tests showed that compared with the retraining trials, young subjects did not require significantly more time to select aiming direction (all delay periods: *p *>* *0.18, *d* < 0.45), while older subjects needed significantly more selection time for all delay periods >10 s (all cases: *p *<* *0.0001, *d* > 1.35).

## Discussion

### Influence of aging on the interaction of implicit and explicit learning mechanisms

Although natural aging is closely associated with learning and memory impairments (Seidler et al., 2010; King et al., 2013; Cai et al., 2014), its effect on motor learning has varied. In some cases, older subjects are significantly slower to compensate for perturbations to movement. For example, Fernández-Ruiz et al. (2000) found that older subjects (age range, 50–78 years) required a longer training period to achieve the same level of prism adaptation as younger subjects (age range, 18–24 years). Similarly, in a visuomotor rotation task, Bock and Girgenrath (2006) reported older subjects (age range, 62–79 years) had a slower rate of movement recalibration compared with younger subjects (age range, 21–30 years), which corresponded with reduced performance in a cognitive reaction-time tasks. In contrast, other studies have shown little effect of aging on motor recalibration; young subjects (mean age, 23.1 ± 4.2 years) and older subjects (mean age, 71.1 ± 7.8 years) demonstrated a similar time course of adaptation in a force-field adaptation task (Trewartha et al., 2014). Similarly, Malone and Bastian (2016) showed young subjects (mean age, 22.5 ± 2.6 years) and older subjects (mean age, 52.8 ± 5.8 years) adapted at similar rates in response to perturbations of gait patterns.

In the current study, we examined the respective age-dependent contributions of explicit and implicit learning mechanisms to motor adaptation (Mazzoni and Krakauer, 2006; Heuer and Hegele, 2008, 2014; Lam et al., 2010; Taylor et al., 2014; Bond and Taylor, 2015; Morehead et al., 2015; De Brouwer et al., 2018; Vachon et al., 2020). Although young and older subjects adapted to the feedback rotation at somewhat similar rates (Fig. 2), older subjects consistently exhibited a higher implicit learning contribution (Fig. 3). This is largely consistent with a number of prior studies. For example, Vandevoorde and Orban de Xivry (2019) recently examined visuomotor learning in young subjects (mean age, 22.8 ± 2.9 years) and older subjects (mean age, 66.8 ± 4.7 years). Implicit learning was not impaired in older subjects, while there was a deficit in the use of cognitive strategies. Additionally, in the study by Malone and Bastian (2016) older subjects demonstrated lower adaptation curves when challenged with a distraction in the form of visual and auditory stimuli, suggesting that diverted attention hindered the ability to access and implement explicit learning strategies. Next, Buch (2003) implemented a visuomotor adaptation task in which the perturbation either increased in 11.25° increments every 45 trials (gradual) or were immediately exposed to a 90° perturbation (sudden). The hypothesis was that a gradual rotation would primarily entail implicit strategies, since subjects would purportedly not be consciously aware of the perturbation, while the sudden rotation was assumed to require more focus on declarative learning. Older subjects demonstrated larger errors than younger subjects during sudden adaptation, suggesting impaired explicit strategies. In contrast, both age groups exhibited similar performance in the gradual rotation task, suggesting comparable use of implicit learning mechanisms during adaptation. Finally, Wolpe et al. (2020) examined visuomotor adaptation in young subjects (age range, 18–45 years), middle-aged subjects (age range, 46–65 years), and older subjects (age range, 66–89 years), and found an age-related decline in motor adaptation. Wolpe et al. (2020) associated this deficit with an age-related decline in explicit memory systems based on behavioral correlations with (1) explicit memory measures (e.g., the Anna Thompson Story Recall task) and (2) the reductions in gray matter volume. It is important to note that, similar to the majority of previous studies described above, Wolpe et al. (2020) did not directly assess implicit and explicit contributions to the overall adaptation, but inferred the role of explicit mechanisms by secondary tasks and correlation analyses. However, as noted above, our results are largely aligned with these conclusions; older subjects showed less of an explicit aiming contribution during learning (Fig. 2). It would be interesting to expand the findings of Wolpe et al. (2020) to determine the relationship between these identified neural areas and the differences in the short-term temporal stability of learning shown here. For example, the authors found that age-related differences in motor adaptation were associated with gray matter volume reductions in the striatum and prefrontal cortex, but not in the cerebellum. In addition, adaptation was associated with gray matter volume in the hippocampus, a relationship that increased with age. For the older subjects in the current study, this would be consistent with largely intact cerebellar-based implicit learning mechanisms during training, but perhaps impaired short-term memory retention because of an age-based deficit of the hippocampus and associated connections (Albouy et al., 2008; Doyon et al., 2009; Addante, 2015).

The greater reliance on implicit learning by older subjects in the current results may be because of a disadvantage in applying explicit knowledge (Light and Singh, 1987; Durkin et al., 1995). Recently, Miyamoto et al. (2020) found that although visuomotor adaptation involves similar levels of implicit and explicit strategies, the two learning mechanisms act antagonistically, with implicit learning compensating for the variability caused by the explicit movement strategy. Our results may reflect this trade-off; although older subjects demonstrated increasing levels of explicit learning as training progressed, the level of overall adaptation remained relatively consistent. As a result, there was increased use of implicit learning when explicit mechanisms were hindered, as implicit learning may have provided a stability advantage when attempting to maintain overall adaptation. That is, the greater reliance on implicit learning mechanisms by older subjects may be beneficial in countering a possible age-associated increase in explicit strategy-induced performance variability.

### Influence of aging on the temporal stability of implicit and explicit learning mechanisms

Similar to previous studies of age-related differences in the temporal decay of performance (Loaiza and McCabe, 2013; Trewartha et al., 2014; Malone and Bastian, 2016), all subjects experienced a decrease in the overall learning with time. This is consistent with prior adaptation studies that demonstrated lower aftereffects either as a function of time or over consecutive no-feedback trials (Galea et al., 2011; Kitago et al., 2013; Taylor et al., 2014; Zhou et al., 2017). However, the pattern of temporal stability differed between the subject groups because of an age-related difference in the rate of the decay of implicit learning—exponential for young subjects and linear for older subjects. The differences in decay rate between the young and older subject groups suggests that there would continue to be significant age-related differences in retention at longer delay periods, since the retention in younger subjects had reached asymptote after ∼30 s. It is important to note that these results point to an age-related deficit in the short-term temporal stability, which appears in contrast to the previous finding that older and younger subjects had similar retention of implicit and overall adaptation over a 1 min break during learning (Vandevoorde and Orban de Xivry, 2019). However, Vandevoorde and Orban de Xivry (2019) assessed this retention after a single time point, whereas we were interested in how this retention systematically changed over time. In addition, even in our study this single time point shows similar levels of decay between the two groups (Fig. 7, near the 50 s time point); extending the range beyond a minute (>90 s) provides a full view of the difference in the decay rates. Furthermore, we assessed the age-based difference in stability after asymptotic levels of learning had been achieved and maintained (through retraining), not during training as in this previous study. Because of the short timescale studied (seconds to 1.5 min), the current results do not contradict the observed age-related retention deficits of explicit memory functions for nonmotor tasks over longer time periods (Naveh-Benjamin et al., 2003; Kessels et al., 2007). Finally, prior studies that observed a decrease of adaptation over consecutive movement trials examined a combination of movement-dependent and time-dependent decay, rather than strictly time-dependent changes—a difference likely involving distinct neural mechanisms (Kitago et al., 2013).

There is ample evidence that short-term motor adaptation involves different learning mechanisms operating along multiple timescales (Smith et al., 2006; Körding et al., 2007; Criscimagna-Hemminger and Shadmehr, 2008; Joiner and Smith, 2008; Mawase et al., 2014; Inoue et al., 2015; McDougle et al., 2015; Alhussein et al., 2019). Sing et al. (2009) hypothesized that there is an interaction of temporally labile and temporally stable learning mechanisms (represented by fast and slow learning processes; Smith et al., 2006) that interact during adaptation. The temporally labile fast learning process decreases with the passage of time (passive decay), while the temporally stable slow component is not significantly affected with the passage of time. The slower decay of adaptation for young subjects may represent the relative stability of the temporally labile fast process, while the greater decay for older subjects may reflect the combined fading of both the slow and fast learning mechanisms. However, because adaptation to visual manipulations involves explicit cognitive strategies, we should note that state-space models that reflect fast and slow learning processes fail to capture some aspects of this type of motor recalibration (Zarahn et al., 2008). Additional studies must be conducted to develop a computational framework that fully captures these age-based behavioral results in terms of temporally labile and temporally stable learning mechanisms.

Our results presented in Figure 5 suggest that during early training the increase of the explicit aiming was related to the increase of the selection time; with longer selection time, subjects in both groups had a larger explicit aim to counter the perturbation. Although demonstrated on a significantly smaller timescale (milliseconds as opposed to seconds/minute), this benefit of increased decision time is observed in previous findings (Haith et al., 2015, 2016) where subjects exhibited significantly greater error on trials with low preparation time compared with trials with high preparation time. Importantly, the results suggest that the time allowed to prepare the motor output differentially influences the explicit and implicit learning components—more time allows for a greater contribution of explicit strategy. The shallower relationship in early training for older subjects suggests that they required more time to form an aiming direction that young subjects were able to formulate in much less time. In addition, all subjects required more time to select an aiming direction as the delay period increased (Fig. 8). However, similar to training (Fig. 4), the older subjects often required twice the amount of time compared with young subjects, suggesting greater uncertainty in forming the explicit plan. This finding is consistent with prior studies (Trewartha et al., 2014; Trewartha and Flanagan, 2016), where older subjects had a longer task reaction time during an explicit memory task. Furthermore, the increased reaction time in the older group for the memory task was found to correspond with reduced explicit memory performance, demonstrating a negative relationship between reaction time and explicit memory. Trewartha and Flanagan (2016) demonstrated similar findings in the context of a weight prediction task. Our results did not show a similar temporal relationship; older subjects were able to maintain aiming accuracy although the selection time increased (Figs. 7, 8). However, in both the training and testing phases, the longer selection time for older subjects may result from uncertainty in the aiming direction, rather than a general difficulty in making the selection. That is, there was no significant change in the explicit aiming at the end of training (Fig. 3) or over the testing phase (Fig. 7), but older subjects continued to require significantly more selection time compared with young subjects.

### Potential application to neurodegenerative disorders

Our experimental paradigm may provide a basis for clinical assessments of implicit and explicit learning and memory deficits. For example, it is well known that normal aging contributes to neurochemical and physiological alterations within the brain, including cortical thinning and decreased hippocampal volume (Nyberg et al., 2012), leading to reduced working and episodic memory (Park et al., 2002; Rönnlund et al., 2005; Malouin et al., 2010; Sauzéon et al., 2016), pair association (Light et al., 2004; Old and Naveh-Benjamin, 2008), and decision-making (Cai et al., 2014). There is also evidence that proprioceptive perception and associated processing declines with age (Adamo et al., 2007; Goble et al., 2009; Piitulainen et al., 2018), which likely contribute to deficits in error-based learning and decision tasks. Treatment of neurodegenerative diseases requires a thorough understanding of how these age-related changes in sensory processing and memory stability are distinct from pathologic deficits. For example, Alzheimer’s disease (AD) patients commonly experience memory deterioration and a decreased ability to make judgments and decisions (e.g., the Rey Auditory Verbal Learning Test and verbal fluency tasks; Klimkowicz-Mrowiec et al., 2008). In contrast, implicit learning remains largely intact, specifically in simple motor tasks (e.g., maze test, rotor pursuit, mirror tracing, and serial reaction time; Heindel et al., 1988, 1989; Knopman, 1991; Ferraro et al., 1993; Dick et al., 1995, 2001, 2003; Starkstein et al., 1997; Taylor, 1998; Rouleau et al., 2002). Our findings provide a possible standard to distinguish motor learning and retention deficits accompanying normal aging from those caused by brain disorders that progress with age. One hypothesis is that the overall short-term temporal stability of motor adaptation will be significantly impaired for AD patients because of an abnormal fading of the explicit aiming direction over time, in addition to the reduced ability to store the implicit memory component caused by hippocampal damage. Importantly, age-associated differences in the short-term temporal stability of implicit learning in the current study were observed despite similar performance on the MMSE and TMT. Thus, analyzing the temporal properties of implicit and explicit motor learning within the same behavioral context may provide a novel behavioral assay to determine the potential biomarkers of the associated structural and functional neural changes that accompany neurodegenerative disorders.

### Conclusions

In this study, we examined the differences in the short-term (seconds to 1.5 min) temporal stability of explicit and implicit motor learning following adaptation of arm-reaching movements. We specifically focused on possible variations in the stability because of normal aging. In general, both young and older subjects showed similar overall learning rates of adaptation, and by the end of training there was not a significant difference in the level of explicit aiming and implicit learning between the two groups. Interestingly, over a short-term delay period (from 0 to 90 s), both young and older subjects showed almost complete retention of the explicit aiming achieved during adaptation. However, the temporal change for implicit learning was very different between subject groups. Consistent with previous work, young subjects had an exponential decrease in the implicit learning component. In stark contrast, older subjects had a linear decrease over the same time period. These results demonstrate that within the context of this motor adaptation task, normal aging reduces the short-term stability of implicit motor learning across time, with very little impact on explicit motor learning processes.

## Synthesis

Reviewing Editor: Satu Palva, University of Helsinki Decisions are customarily a result of the Reviewing Editor and the peer reviewers coming together and discussing their recommendations until a consensus is reached. When revisions are invited, a fact-based synthesis statement explaining their decision and outlining what is needed to prepare a revision will be listed below. The following reviewer(s) agreed to reveal their identity: Harri Piitulainen, Jean-Jacques Orban de Xivry. Note: If this manuscript was transferred from JNeurosci and a decision was made to accept the manuscript without peer review, a brief statement to this effect will instead be what is listed below.

This manuscript provides new insights into age-related changes on short-term retention of motor memories. This might have implications for neurological disorders. The authors present results about hand motor learning task to visuomotor rotation of hand trajectory in younger and older individuals. They focus on temporal stability of explicit and implicit learning during repeated tasks (test and learning task alternated with different delays from 0 to 90 s). In general, the experiment is well conducted, and report is well written, but would benefit from condensation and clarification.

Major concerns

The authors study explicit and implicit learning, but they do not explain these concepts or theories in the Introduction at all. Please explain these concepts and explain more clearly how and why your tasks represented/teste these learning mechanisms?

The implicit motor learning task appears as kinesthetic/proprioceptive task/learning. In this task, there is no visual cue during the movement, and thus it reflects more a somatomotor integration learning/adaptation than visuomotor integration. This should be emphasized for the current task(s) and results in Introduction and Discussion. Interestingly, both the proprioceptive perception and cortical processing of the proprioception are impaired with aging, that could partly explain your result of slower learning/processing (e.g. selection time) of the currently used tasks. See:

- Goble et al. 2009. Proprioceptive sensibility in the elderly: Degeneration, functional consequences and plastic-adaptive processes. Neurosci. Biobehav. Rev. 33, 271-278., DOI: 10.1016/j.neubiorev.2008.08.012

- Piitulainen et al. 2018. Cortical Proprioceptive Processing Is Altered by Aging. Front Aging Neurosci. 10:147. DOI: 10.3389/fnagi.2018.00147.

Selection time is very long, there is almost a short-term retention period between each trial when the selection time is 10sec or more. It is very different for some of the older adults who need up to (on average) 20s (15s during the testing phase) to select movement direction. I believe that this is an underestimation as the authors excluded trials with selection time larger than 30s. Yet, these excluded trials will influence the adaptation process because they make the inter-trial time longer for these specific subjects. This will have an influence on total adaptation rate. The fact that the selection time is much larger in older adults is a major problem as these individuals have then an overall longer inter-trial duration, which has an influence on the amount of adaptation. It is also directly influence the estimation of the delay period as this can be up to 200% larger than instructed (30s instead of 10s for selection time of 10s and delay of 10s). The authors should perform their analyses with the actual elapsed time during the delay period rather than the instructed/imposed delay time (in Fig.6, replace delay time with actual elapsed time). Note that the elapsed time could be different for the explicit retention (from end of previous trial to time of selection) and for the implicit retention (from end of previous trial to time of movement start?). One way to proceed who be to use the actual average elapsed time for each age group but it would be even better if there was a way to take into account inter-individual differences in elapsed time as this varies a lot between the participants, especially for the older adults (if not possible on the graph, it should at least be added as co-variate in the statistical analysis).

Statistics of overall adaptation do not fit with what one sees on the figure. Statistics as performed currently are very dependent on the selection of time windows (middle and late). It would be better to perform an unbiased analysis by taking the mean over all the learning period (trials 1-60).

Tiny differences in baseline should be taken into account in the ANOVA as a covariate. The data from the blank trials during baseline are never reported. How accurate were people during baseline in aiming to the selected point? These values should be described and should be taken into account as a co-variate in the analysis (not simply substracting).

There is no statistical results that support the main conclusion of the paper (decay in implicit is different for young and old). Yet, when both implicit and explicit are used in the LMM (line 472), there is a main effect of age group, learning type and delay period and only an interaction between delay time and learning group. In other words, on this basis, one could not conclude that the difference in retention is specific to the implicit component as there is no interaction between learning type, delay period and age group. This statistical analysis does not match with the conclusions of the authors. The rest of this paragraph is describing that there was no difference in explicit retention between age groups but there was one in implicit retention. Yet, only an interaction can tell that this is a true difference. This is the problem reported by Nieuwenhuis et al. 2011. I believe that the absence of significant triple interaction is due to the huge variability in retention values for the group of older adults.

The task has been well validated in younger adults. On most graphs, the authors provide individual datapoints, but there should be more of them. It is also important that they measure the explicit and implicit components of motor adaptation.

Results: Figure 3. Were there any significant differences here? The inter-individual variation is huge, and thus the sample size of the current data is not sufficient. With bigger sample size many of the ns. Results would be significant. Based on the text, the only difference was for older group in middle training period between implicit and explicit training. Please indicate the significant results clearly to the Figures.

The learning task is exhaustively repeated throughout the manuscript. It should be explained as clearly as possible in the Methods section, and then only briefly reminded in the later sections. It would be good to include more details also in the Figure 1 about the task (number of repetitions etc.). This work would also benefit from illustrative Video in the Supplements about the task if possible. Now it took some time to understand the protocol from the text and Figure 1.

The Results are exhausting and could be greatly condensed to improve clarity. I would omit all discussion from the Results (e.g. L321¬-329) and move it to the Discussion only.

There is very little data on the different measures linked to retention (Fig.6). Selection time are provided on Fig.7 but the retention is primarily based on the difference between the last three trials (with visual feedback) before the break and the first trials after the break. Yet, there is currently absolutely no information on these three trial averages and this is required as they have a large influence on the retention parameters. The authors should provide 7 scatterplot graphs (one graph per delay value, on each graph: one point per participant, color-coded for age group) with average adaptation in the three movements (average across the 4 trials for that delay) on the x-axis vs. the average amount of adaptation on the retention trial without visual feedback (average across the 4 trials for that delay). The goal is to present the data from the adaptation trials during the testing phase and from the retention trials for each individual participant and each delay. This is currently missing in the paper.

The adaptation behavior during the testing phase should be described and illustrated for both age groups. To account for potential differences in adaptation before retention, I would like the authors to perform their analyses on relative retention parameters (adapt-retained/adapt) rather than on the absolute difference as they did here.

Influence of baseline: the angle decay over time (ADoT) is computing by subtracting the baseline-corrected adaptation measured during retention trials (R-B) from the average of non-baseline-corrected adaptation trials before the delay (A). By doing so, the researchers are essentially adding the baseline correction to the angle decay observed during the delay period (ADoT = (A-R) + B).

The values presented on Lines 419-420 seem inconsistent with the values presented on Fig.6. For the young group, there seems to be a small increase in adaptation during the 0s break (from 43.47{degree sign} to 44.03{degree sign}). Yet, the average of total adaptation for the 0s delay on the left panel of Fig.6 is below zero while it should be above it. How is the value on line 433 (-0.27{degree sign}) is obtained?

Minor comments

Conclusions are missing from the end of the Discussion, but are more or less mentioned in the 1st paragraph of Discussion. I would move the primary Conclusions to a specific Conclusions section in the end of the Discussion.

Please add titles to the panels of Figures in addition to panel labels. It would be much easier to understand what is presented in each panel.

It would be more correct to call ‘elderly’ group as ‘older’ group

How does Fig.5 contribute to our understanding of possible differences in short-term retention between young and old adults. Furthermore, I don’t agree that this is consistent with Haith et al. (lines 389-391) given that their low and high PT was in the order of a few 100ms and not several seconds. In addition, it does not make sense that the slope was steeper for older adults early in the adaptation period.

Vandevoorde and Orban de Xivry 2019 reported no difference in short-term retention between young and old participants over a one-minute break for total adaptation and for implicit adaptation only (isolated by design)

Why does the effect of group reported on line 429 does not match the effect of group on line 476? The model is different but should not the statistical results be the same because total adaptation = exp.

## Author Response

Dear Dr. Palva:

We have revised our manuscript titled “Normal aging reduces the short-term temporal stability of implicit, but not explicit, motor learning following visuomotor adaptation “ (eN-TNWR-0527-20X). We thank the Reviewers for their very careful and insightful comments. Our manuscript is much improved based on their suggestions. We have addressed each of the raised concerns marked in red below. Specifically, in the revised manuscript we now use the actual elapsed time in our quantification of learning decay and statistical analysis, include the suggested analysis directly comparing the learning on probe trials to the previous re-training trials, and have significantly reduced the redundancy in the Methods and Results. Changes are marked in red throughout the redline version of the revised manuscript. Please note that the lines where the revised text can be found corresponds to the redline document. We believe that we have fully addressed the Reviewers’ concerns, and as a result, we now have a much improved manuscript that we respectfully resubmit for your consideration.

Sincerely,

Guneet Bindra, Rylee Brower, Ryan North, Weiwei Zhou and Wilsaan M. Joiner

Synthesis Statement for Author (Required):

This manuscript provides new insights into age-related changes on short-term retention of motor memories. This might have implications for neurological disorders. The authors present results about hand motor learning task to visuomotor rotation of hand trajectory in younger and older individuals. They focus on temporal stability of explicit and implicit learning during repeated tasks (test and learning task alternated with different delays from 0 to 90 s). In general, the experiment is well conducted, and report is well written, but would benefit from condensation and clarification.

We thank the Reviewers for the careful and insightful review of our manuscript. We have addressed these concerns below.

Major concerns

1. The authors study explicit and implicit learning, but they do not explain these concepts or theories in the Introduction at all. Please explain these concepts and explain more clearly how and why your tasks represented/teste these learning mechanisms?

In the Introduction we have now added a brief explanation of implicit and explicit learning where we describe the current understandings of these learning components. In addition, we connect these learning mechanisms to the visuomotor rotation adaptation paradigm we implemented, in order to demonstrate how the task accounts for both learning components (Lines 58-70):

"One notable task involves visuomotor rotation (VMR), in which individuals adapt to a visual feedback perturbation (i.e., a rotation of the feedback trajectory, Krakauer et al. 1999; Redding et al. 2005). This adaptation has been shown to involve concurrent explicit and implicit learning mechanisms (Taylor et al. 2014). Implicit learning mechanisms utilize sensory-prediction errors to update the internal forward model, likely mediated by the cerebellum on a subconscious level (Synofik et al. 2008; Tseng et al. 2007). Additionally, explicit processes also contribute to the adaptation to the rotation of the feedback through conscious efforts, minimizing the experienced target errors (Bond et al. 2015; Mazzoni and Krakauer 2006). Thus, the VMR paradigm provides a method for assessing both explicit and implicit components of motor adaptation; the explicit component of learning is determined through an aiming report made by the subject, while the implicit learning is based on subtracting this planned movement direction from the actual movement trajectory (Taylor et al. 2014; Morehead et al. 2015).”

2. The implicit motor learning task appears as kinesthetic/proprioceptive task/learning. In this task, there is no visual cue during the movement, and thus it reflects more a somatomotor integration learning/adaptation than visuomotor integration. This should be emphasized for the current task(s) and results in Introduction and Discussion. Interestingly, both the proprioceptive perception and cortical processing of the proprioception are impaired with aging, that could partly explain your result of slower learning/processing (e.g. selection time) of the currently used tasks. See:

- Goble et al. 2009. Proprioceptive sensibility in the elderly: Degeneration, functional consequences and plastic-adaptive processes. Neurosci. Biobehav. Rev. 33, 271 278., DOI: 10.1016/j.neubiorev.2008.08.012

- Piitulainen et al. 2018. Cortical Proprioceptive Processing Is Altered by Aging. Front

Aging Neurosci. 10:147. DOI: 10.3389/fnagi.2018.00147.

We thank the Reviews for this comment. However, as noted in the Methods and Materials, there is a visual cue (the target), full visual feedback of the cursor on the training and re -training trials, and additional endpoint feedback at the end of the movement on these respective trials. If we

have misinterpreted the comment, we will gladly address this concern. That said, in the revised manuscript we now include these age-related deficits in proprioceptive perception and the cortical processing of this information in the Discussion. Specifically, we include this information when discussing how these issues that accompany natural aging must be taken into account when distinguishing motor learning and retention deficits associated with normal aging to those caused by brain disorders (Lines 670-680):

"Our experimental paradigm may provide a basis for clinical assessments of implicit and explicit learning and memory deficits. For example, it is well known that normal aging contributes to neurochemical and physiological alterations within the brain, including cortical thinning and decreased hippocampal volume (Nyberg et al. 2012), leading to reduced working and episodic memory (Park et al. 2002; Rönnlund et al. 2005; Malouin et al. 2010; Sauzéon et al. 2016), pair association (Light et al. 2004; Old and Naveh-Benjamin 2008), and decision-making (Cai et al.

2014). There is also evidence that proprioceptive perception and associated processing declines with age (Adamo et al. 2007; Goble et al. 2009; Piitulainen et al. 2018), which likely contribute to deficits in error-based learning and decision tasks. Treatment of neurodegenerative diseases requires a thorough understanding of how these age-related changes in sensory processing and memory stability are distinct from pathological deficits.”

3. Selection time is very long, there is almost a short-term retention period between each trial when the selection time is 10sec or more. It is very different for some of the older adults who need up to (on average) 20s (15s during the testing phase) to select movement direction. I believe that this is an underestimation as the authors excluded trials with selection time larger than 30s. Yet, these excluded trials will influence the adaptation process because they make the inter-trial time longer for these specific subjects. This will have an influence on total adaptation rate. The fact that the selection time is much larger in older adults is a major problem as these individuals have then an overall longer inter-trial duration, which has an influence on the amount of adaptation. It is also directly influence the estimation of the delay period as this can be up to 200% larger than instructed (30s instead of 10s for selection time of 10s and delay of 10s). The authors should perform their analyses with the actua l elapsed time during the delay period rather than the instructed/imposed delay time (in Fig.6, replace delay time with actual elapsed time). Note that the elapsed time could be different for the explicit retention (from end of previous trial to time of selection) and for the implicit retention (from end of previous trial to time of movement start?). One way to proceed who be to use the actual average elapsed time for each age group but it would be even better if there was a way to take into account inter-individual differences in elapsed time as this varies a lot between the participants, especially for the older adults (if not possible on the graph, it should at least be added as co-variate in the statistical analysis).

We agree with the Reviewers and thank them for these very helpful suggestions. We also apologize for the oversight in not clearly presenting the analysis of the selection time ; as pointed out, the selection time should be included in the change in adaptation with the imposed delay and in the statistical analysis.

On the first point, the reason we excluded the trials with a selection time larger than 30s is that all of these trials were at beginning of the training session when subjects first experienced the visuomotor rotation and were initially becoming familiar with how to make the selection using the knob (2.1% of training trials with selection time large than 30s occurred for the elderly group and

0.6% for the young group. All of these trials were in the first half of the training phase: trials 1-30). Based on our statistical analysis, selection times larger than 30s were outliers (greater than 3 times of the standard deviation of the average selection time for each respective group). Subjects

in both groups did not make selections with larger than 30s for trials in the second half of the training session (trials 31-60) and the entire testing session. Thus, excluding these trials did not affect the main findings of the study. We have clarified these points in the revised manuscript (Lines 234-243):

"Trials that had very slow/fast movement speeds (peak speed < 0.22 m/s or > 0.5 m/s) and long selection time (selection time > 30 seconds) were excluded (∼3% of total trials for the young group and ∼4.5% of total trials for the older group). The reason we excluded the trials with a selection time larger than 30s is that all these trials were at beginning of the training session when subjects first experienced the visuomotor rotation and were becoming familiar with rotating the knob and making an aiming selection. There were 2.1% of training trials with a selection time larger than

30s for the older group and 0.6% for the young group. All of these trials were in the first half of the training phase; none of the subjects made a selection with a time larger than 30s for trials in the second half of the training session and the entire testing phase.”

On the second point, we agree with the Reviewers that using the actual elapsed time (the imposed delay and the selection time) is a much better way to characterize the decay for both the explicit and implicit learning. In the revised manuscript we have described this in the Methods (Lines 206-

213):

"Due to the variance in the selection time and movement duration across subjects, we used the actual elapsed time to define the delay for explicit and implicit learning instead of the imposed 7 delay periods in the experiments to better capture the influence of the delay for each learning component. Thus, the delay for the explicit learning component was defined as the time interval from the end of previous trial to the time when the selection was made. The delay for the implicit learning component was defined as the time interval from the end of the previous trial to the movement onset. (Note the actual elapsed time was always slightly longer for the implicit learning component.)"

The temporal retention of the explicit and implicit learning in now in terms of actual elapsed time in Figure 7A and B (see below). (Lines 431-436):

"Thus, the overall temporal retention for both groups decayed significantly as the actual delay period increased (actual delay at 0s: 2.47s {plus minus} 0.19s, 5s: 8.01s {plus minus} 0.23s, 10s: 13.19s {plus minus} 0.27s, 20s:

23.11s {plus minus} 0.30s, 30s: 33.32s {plus minus} 0.24s, 50s: 53.71s {plus minus} 0.33s, 90s: 93.07s {plus minus} 0.65s for the young group;

0s: 4.01s {plus minus} 0.52s, 5s: 10.55s {plus minus} 0.81s, 10s: 16.14s {plus minus} 0.75s, 20s: 26.74s {plus minus} 0.88s, 30s: 36.81s {plus minus}

0.86s, 50s: 55.16s {plus minus} 0.1.19s, 90s: 96.91s {plus minus} 0.96s for the older group).”

We also modified the corresponding statistical analysis (taking into account the actual elapsed time as a covariate, Lines 247-250):

"To examine the potential difference in the temporal retention of the learning between the two groups, we fitted the absolute angular decay and the relative decay percentage (see above) using a LMM with fixed effects of group and learning type, random effect of subjects, and actual elapsed time as a covariate.”

This greatly improved our conclusions on the role the different learning mechanisms played during the decay of adaptation (Lines 461-469):

"Significance was found for all three fixed effects (F(1, 488.16) = 49.35, P < 0.0001 for actual elapsed time, F (1, 484.68) = 156.10, P = 0.0018 for learning type and F (1, 75.87) = 10.52, P =

0.0018 for group) and interaction among the three fixed effects (F(1, 484.89) = 9.62, P = 0.002). Post hoc tests showed that for both groups, implicit learning decay was significantly larger than the explicit learning decay over time (tratio(484) = 3.02, P = 0.0026 for the young group; tratio(486)

= 7.19, P < 0.0001 for the older group). However, there was no significant difference in the temporal angular decay of explicit aiming between the two groups (tratio(486) = 0.18, P > 0.85) and temporal angular decay of the implicit learning for the older group was significantly larger than the decay of the young group (tratio (484) = -4.17, P < 0.0001).”

Lines 486-493:

"The same LMM with random effect of subjects was performed to examine the effects of actual elapsed time, learning type, and age group on the relative temporal decay of the explicit and implicit learning. Significance was found for the interaction among the three fixed effects (F(1,

484.89) = 4.80, P = 0.029). Post hoc tests revealed the same results that relative retention of the explicit learning were not significantly different between the two groups (tratio(486) = 0.36, P > 0.71) and the relative temporal decay of the implicit learning for the older group was significantly larger than the decay of the young group (tratio (484) = -2.69, P = 0.0074).”

4. Statistics of overall adaptation do not fit with what one sees on the figure. Statistics as performed currently are very dependent on the selection of time windows (middle and late). It would be better to perform an unbiased analysis by taking the mean over all the learning period (trials 1-60).

We agree with the Reviewers that the statistics on the adaptation levels during training are somewhat dependent on the selection of the windows (placement and size). However, it is important to note that our goal was to show the progression of the different learning components and the selection time for both groups at different points during the training. We chose the periods (early, middle, and late) based on the number of trials and made sure the window sizes were consistent for all comparisons/analyses. Unfortunately, taking the mean over the entire learning period would obscure the ability to observe any progressive change in performance. We therefore maintained the selection time windows, but now provide more context for these periods (Lines

161-166):

"We defined three periods during training in order to compare learning between groups (early training: the first 5 training trials (1-5), middle training: the middle 5 trials of training (28-32) and late training: the last 5 training trials (56-60)). Note that these periods were used to show the progression in behavior throughout training and were based on the total number of training trials. Also, the results are similar for different window sizes (3 or 7 trials).”

As suggested we also report the mean values for the different learning components and overall learning (Lines 285-292):

"We first evaluated the mean adaptation over the training period for both groups. Throughout the entire training period, the subjects in the young group had a significantly higher explicit learning level (17.73o {plus minus} 3.40o for the young group and 11.56o {plus minus} 3.6o for the older group; two-tailed unpaired t-test, P > 0.022, d = 0.76) and a similar implicit learning level compared to the subjects in the older group (16.35o {plus minus} 3.43o for the young group and 18.65o {plus minus} 3.64o for the older group; two-tailed unpaired t-test, P = 0.26, d = 0.37), leading to significantly higher overall adaptation for the young group (34.07o {plus minus} 4.55o for the young group and 30.20o {plus minus} 4.68o for the older group; two-tailed unpaired t-test, P > 0.023, d = 0.75).”

5. Tiny differences in baseline should be taken into account in the ANOVA as a covariate. The data from the blank trials during baseline are never reported. How accurate were people during baseline in aiming to the selected point? These values should be described and should be taken into account as a co-variate in the analysis (not simply substracting).

We agree with the Reviewers that the baseline should be evaluated and reported for both groups. This information was provided in the initial submission under Baseline Performance. We observed no difference in reach angle between the groups, and the learning components were not significantly different from zero (Lines 264-279):

"All subjects practiced selecting the aiming direction and reaching to the targets during the baseline block with 20% blank no-visual feedback trials (a total of 8 blank movements). During the baseline trials, all participants in both groups moved the cursor directly to the target, and the reach angles were not significantly different from each other (young: 0.45o {plus minus}

0.23o, older: 0.47o {plus minus} 0.29o; two-tailed unpaired t-test, P = 0.97, d = 0.012) and not significantly

different from 0o (two-tailed one sample t-test, P = 0.16, d = 0.37 for the young group and P =

0.12, d = 0.44 for the older group). Participants demonstrated no explicit aiming or implicit learning during this block, as expected (explicit: young: -0.029o {plus minus} 0.046o, older: -0.031o {plus minus} 0.10o; two-tailed one sample t-test, P = 0.53, d = 0.14 for the young group and P = 0.75, d = 0.073 for the older group; implicit: young: 0.48o {plus minus} 0.24o, older: 0.50o {plus minus} 0.32o; two-tailed one sample t-test, P = 0.06, d = 0.45 for the young group and P = 0.13, d = 0.36 for the older group). Additionally, there were no significant differences between the explicit and implicit components of adaptation in both groups (An analysis using LMM with fixed effects of group and learning type and random effect of subjects: F(1, 74) = 0.0012, P = 0.97 for group, F(1, 74) = 0.35, P = 0.12 for learning type and F(1, 74) = 0.0021, P = 0.96 for the interaction between the two fixed effects).”

When we included the differences in baseline as a covariate in the analysis we saw no difference in the statistical results. Thus, due to a lack of any differences in baseline we decided not to include these measures as a covariate in the revised manuscript.

6. There is no statistical results that support the main conclusion of the paper (decay in implicit is different for young and old). Yet, when both implicit and explicit are used in the LMM (line

472), there is a main effect of age group, learning type and delay period and only an interaction between delay time and learning group. In other words, on this basis, one could not conclude that the difference in retention is specific to the implicit component as there is no interaction between learning type, delay period and age group. This statistical analysis does not match with the conclusions of the authors. The rest of this paragraph is describing that there was no difference in explicit retention between age groups but there was one in implicit retention. Yet, only an interaction can tell that this is a true difference. This is the problem reported by Nieuwenhuis et al. 2011. I believe that the absence of significant triple interaction is due to the huge variability in retention values for the group of older adults.

We agree with the Reviewers that some of reported statistics in the initial submission were problematic for the exact reasons outlined above. Based on the suggestion above (see comment

3), in the revised manuscript we now perform the analyses with the actual elapsed time instead of the imposed delay periods. We took the inter-individual differences in actual elapsed time into

consideration and set elapsed time as a covariate in our linear mixed -effect models. We also

assessed the relative retention level of the learning (compared to the average adaptation at the end of preceding re-training trials, see comment 12 below). These new and modified analyses have provided much stronger evidence to support our main conclusions.

7. The task has been well validated in younger adults. On most graphs, the authors provide individual datapoints, but there should be more of them. It is also important that they measure the explicit and implicit components of motor adaptation.

For all relevant figures (Figures 3, 4 and 8) we verified that all individual data points are present in order to show the individual differences along with the average statistics. Note that there may be some substantial overlap of individual data points, obscuring an accurate account. If we have misinterpreted this comment and/or there remains an issue with the revised figures, we will gladly fix any remaining concerns.

8. Results: Figure 3. Were there any significant differences here? The inter-individual variation is huge, and thus the sample size of the current data is not sufficient. With bigger sample size many of the ns. Results would be significant. Based on the text, the only difference was for older group in middle training period between implicit and explicit training. Please indicate the significant results clearly to the Figures.

We agree with the Reviewers. In the revised manuscript significance is now reported in all figures. We apologize that this information was missing in the initial submission. Also, we should note that the focus of our study was to examine and quantify the temporal decay of these different components of learning (along with the selection time), which the current sample size is sufficient. Although there is variability during the training phase, in our analysis of the selection time and learning decay (explicit, implicit and overall) we normalize within each subject which strengthens our overall analysis. We are especially thankful for the analysis suggestions (e.g., taking into account the actual elapsed time and examining the relative retention parameters) that greatly strengthen our conclusions.

9. The learning task is exhaustively repeated throughout the manuscript. It should be explained as clearly as possible in the Methods section, and then only briefly reminded in the later sections. It would be good to include more details also in the Figure 1 about the task (number of repetitions etc.). This work would also benefit from illustrative Video in the Supplements about the task if possible. Now it took some time to understand the protocol from the text and Figure 1.

We understand the Reviewers’ comment. Our intent was to be as thorough as possible and provide information on the task if the reader only focused on the Results or Discussion sections of the manuscript. That said, throughout the Results and Discussion in the revised manuscript we have significantly modified and removed sections that explained the task. Also, in the Figure 1 description, we have added more information regarding the organization of the task, number of trials in each block, and the overall design of the experimental apparatus (Lines 908-929):

"Figure 1. Experimental design and task structure. (A) The experimental setup consisted of a LCD monitor that displayed targets and visual feedback of the hand, a digitizing tablet, and a PC that controlled the paradigm. The monitor was 10 inches above the digitizing tablet. The midline of the subject was aligned with the center of the tablet and monitor. (B) Depiction of the explicit aim selection which consisted of a circle centered at the start location (gray filled circle). The movement goal is represented by the red filled circle, and a yellow filled circle represents the selection marker. This circle appeared 10 cm from the starting position. Subjects turned a knob at the beginning of each trial to move the yellow marker around the circle and indicate their explic it aiming direction. (C) The sequence of training and testing the subjects. After 20 familiarization trials, the subjects underwent 40 baseline trials where the cursor followed the hand position. This was followed by 60 training trials, during which a +45{degree sign} (clockwise) or -45{degree sign} (counterclockwise)

visuomotor rotation of the cursor movement feedback was applied. During the testing phase, each subject experienced a delay period (0, 5, 10, 20 30, 50, and 90 seconds). This was followed by a single probe trial, and then 5-7 re-training trials before the next delay period was tested. This pattern was repeated for a total of 4 probe movements for each delay duration. (D) Depiction of hand and cursor movements during baseline, and early and late training. During baseli ne the cursor and hand paths were aligned. When the visuomotor rotation was applied, there was a rotational offset between the cursor and hand path. Late in training subjects adjusted the hand path to guide the cursor to the target. (E) A representation of the explicit and implicit components of overall adaptation. Explicit aiming was measured as the angle difference between the aiming direction and the target, while implicit adaptation was defined as the angle difference between reach direction and aiming direction.”

Although we do not provide a video of the task, we hope that the improved description of the task setup and experimental paradigm have addressed any ambiguity noted in the initial submission.

10. The Results are exhausting and could be greatly condensed to improve clarity. I would omit all discussion from the Results (e.g. L321¬-329) and move it to the Discussion only.

Based on this comment we have tried to streamline the Results section. First, we omitted sentences and phrases within the Results section that were repetitive, including sections that explained the task. We condensed the section by re-structuring and editing some of the sentences for clarity. We also moved certain parts of the Results, such as the discussion of Figure 5 (now lines 642-667), to the Discussion. Finally, we have removed the summary of the mean values and corresponding standard error ranges for Figures 7 and 8. This information can be found in the statistics summary. On the last point (mean values and standard error), if the Reviewers and editor feel that this is necessary information we will gladly include them again in the manuscript, but we thought these details may be excessive in light of the comment above.

11. There is very little data on the different measures linked to retention (Fig.6). Selection time are provided on Fig.7 but the retention is primarily based on the difference between the last three trials (with visual feedback) before the break and the first trials after the break. Yet, there is currently absolutely no information on these three trial averages and this is required as they have a large influence on the retention parameters. The authors should provide 7 scatterplot graphs (one graph per delay value, on each graph: one point per participant, color-coded for age group) with average adaptation in the three movements (average across the 4 trials for that delay) on the x-axis vs. the average amount of adaptation on the retention trial without visual feedback (average across the 4 trials for that delay). The goal is to present the data from the adaptation trials during the testing phase and from the retention trials for each individual participant and each delay. This is currently missing in the paper.

We agree with the Reviewers and now show this supporting data. A new figure (Figure 6) was added following this suggestion to illustrate the relationship between the adaptation level in the last 3 retraining trials and retention on the delay probe trials for explicit aiming, implicit learning and overall learning:

With a corresponding figure legend (Lines 968-976):

Figure 6. Retention of learning with respect to previous re-training trials. The amount of learning (aiming and reaching angle) on the probe trial is plotted against the amount of learning on the previous re-training trials for (A) explicit aiming, (B) implicit learning and (C) overall learning for the seven different delays (each respective column). The individual data for each subject are represented by the green circles (young subjects) and purple squares (older subjects). The group mean in each panel is depicted by the larger filled symbols for each respective group, and the shaded ellipses represent the 95% confidence interva ls. Horizontal and vertical dashed lines show the maximum VMR perturbation (45o) and the diagonal dashed line is the unity line.

The interpretation of this analysis is now included in the Results section (Lines 403-414):

"We first assessed retention of explicit aiming, and implicit and overall learning by comparing the aiming angles and reaching angles on the non-visual feedback probe trials to the average aiming and reaching angles over the last three re-training trials (see Materials and Methods). W e plotted the former as a function of the latter for each individual probe trial in Figure 6. For the explicit learning (Figure 6A), the majority of the data points for both groups were on the diagonal unity lines for the different delay periods, indicating no obvious decay over time (note the mean values in each panel are on the unity lines). For the implicit learning and overall learning (Figure 6B and C), most data points started to fall below the unity line as the delay period increased. Also, the data points for the older group (purple squares) deviated further from the diagonal unity lines than those for the young group (green circles), suggesting greater decay with time (again, the mean values in these panel are below the unity line, with the data for the older group below the young).”

12. The adaptation behavior during the testing phase should be described and illustrated for both age groups. To account for potential differences in adaptation before retention, I would like the authors to perform their analyses on relative retention parameters (adapt-retained/adapt) rather than on the absolute difference as they did here.

We agree and thank the Reviewers for these suggestions. In the revised manuscript we have now described these analyses in the Methods (Lines 198-205):

"For each time delay, retention of learning was quantified by comparing the angular deviation on the no-visual feedback probe trials to the average angular deviation at the end of the preceding re-training trials (the last three trials of the 5-7 re-training sequence). The absolute retention (difference in angular deviation between the probe trial and the average on the re-training trials) and relative retention (percentage of the adaptation retained: (probe trial angle - average angle on the re-training trials) / average angle on the re-training trials) were used to examine the decay of the two learning components and overall learning over the delay periods.”

In addition, the analysis of the relative retention was added in the Results (Lines 470-493):

"To better understand the temporal decay for the two learning components, we also measured the relative retention compared to the end of the preceding re-training trials (Figure 7C and D). The relative retention of the overall learning and explicit learning for both groups follow the same trends as the absolute angular retention shown in Figure 7A and B. Both groups had similar relative retention of the explicit learning, and the relative overall learning decay for the older group was significantly larger than that for the young group. For actual elapsed time up to 100s, the average decay of the overall learning for the older group (15%) was about twice of the decay for the young group (8%). One interesting result we observed was that the relative decay of th e implicit learning for both groups was about twice that of the relative decay of their overall learning. This difference is due to dividing the absolute angular decay by the implicit learning levels at the end of the re-training trials which accounted for about 50% of the overall learning (i.e., both explicit and implicit learning contributed about 50% to the overall learning for both groups at the end of training, Figure 2 and 3). Thus, the relative temporal decay of the implicit learning was actually greater than the relative decay of the overall learning for both groups due to the stability of the explicit aiming (For the young group, time constant: 16.76 {plus minus} 23.44s for the implicit decay and

17.38 {plus minus} 23.84s for the overall decay; for the older group, slope: -0.26 {plus minus} 0.095 for the implicit decay and -0.14 {plus minus} 0.052 for the overall decay). The same LMM with random effect of subjects was performed to examine the effects of actual elapsed time, learning type, and age group on the relative temporal decay of the explicit and implicit learning. Significance was found for the interaction among the three fixed effects (F(1, 484.89) = 4.80, P = 0.029). Post hoc tests revealed the same results that relative retention of the explicit learning were not significantly different between the two groups (tratio(486) = 0.36, P > 0.71) and the relative temporal decay of the implicit learning for the older group was significantly larger than the decay of the young group (tratio (484)

= -2.69, P = 0.0074). “

Finally, the corresponding results are now shown in Figure 7C and D.

With a corresponding figure legend (Lines 978-993):

"Figure 7. Temporal decay curves. The decay of implicit, explicit and overall learning as a function of the actual elapsed time is shown for (A and C) young and (B and D) older subjects. The actual elapsed time is the time interval from the end of previous trial to the time when the selection was made for the explicit learning component, and the time interval from the end of previous trial to the movement onset for the implicit learning component. Note the actual elapsed time was always slightly longer for the implicit learning component. The absolute retention (angular deviation) and relative retention (percentage of the adaptation at the end of preceding re-training trials) are shown on the top and bottom row, respectively. The mean value for each respective measure (overall learning: light blue symbols, explicit aiming: red symbols, implicit learning: dark blue symbols) on the probe trial is plotted for the actual elapsed time for each respective delay period (0, 5, 10, 20, 30, 50, and 90 seconds). Each panel includes the best fit function (Panels A and C: exponential for young subjects, Panels B and D: linear for older subjects) for the decay of overall adaptation and implicit learning. Explicit learning for each group was best described by a linear function. Vertical error bars represent SEM in the respective measures of decay, and horizontal error bars represent SEM in the actual elapsed time.”

The new results based on the relative retention have indeed strengthen our conclusions and we thank the Reviewers again for this suggestion.

13. Influence of baseline: the angle decay over time (ADoT) is computing by subtracting the baseline-corrected adaptation measured during retention trials (R-B) from the average of non- baseline-corrected adaptation trials before the delay (A). By doing so, the researchers are essentially adding the baseline correction to the angle decay observed during the delay period (ADoT = (A-R) + B).

This was a mistake in our description of the analysis and we apologize for the confusion. The angle decay over time was computed by subtracting the non-baseline-corrected adaptation measured during retention probe trials from the average of the non-baseline-corrected re- adaptation trials before the delay. We have corrected the mistake and made this point clear in the revised manuscript (Lines 200-206):

"The absolute retention (difference in angular deviation between the probe trial and the average on the re-training trials) and relative retention (percentage of the adaptation retained: (probe trial angle - average angle on the re-training trials) / average angle on the re-training trials) were used to examine the decay of the two learning components and overall learning over the delay periods. These measures of retention were a direct comparison before and after the delay; there was no need for a baseline correction.”

14. The values presented on Lines 419-420 seem inconsistent with the values presented on Fig.6.

For the young group, there seems to be a small increase in adaptation during the 0s break

(from 43.47{degree sign} to 44.03{degree sign}). Yet, the average of total adaptation for the

0s delay on the left panel of Fig.6 is below zero while it should be above it. How is the value

on line 433 (-0.27{degree sign}) is obtained?

We agree with the Reviewers and apologize for the confusion. Although these values have been removed from the Results section in order to reduce its length, this was a miscalculation on our part. We have corrected this in the revised manuscript (Lines 418-422):

"For angular decay (Figure 7A), at the delay of 0s, the amount of compensation for the VMR was not significantly different from the amount of overall recalibration during re -training for both groups (re-training: 44.94o {plus minus} 0.66o and 0s: 44.97o {plus minus} 0.81o, two-tailed paired t-test P > 0.94, d =

0.0065 for the young group; re-training: 42.29o {plus minus} 0.91o and 0s: 41.47o {plus minus} 1.22o, two-tailed paired t- test P > 0.48, d = 0.17 for the older group).”

In addition, we have modified this analysis based on the Reviewers’ suggestions and made sure that any errors in these calculations have been corrected.

Minor comments

1. Conclusions are missing from the end of the Discussion, but are more or less mentioned in

the 1st paragraph of Discussion. I would move the primary Conclusions to a specific

Conclusions section in the end of the Discussion.

As suggested, in the revised manuscript we moved the first paragraph of Discussion to the end so that it now is the Conclusion (Lines 700-712):

"In this study we examined the differences in the short-term (seconds to 1.5 minutes) temporal stability of explicit and implicit motor learning following adaptation of arm reaching movements. We specifically focused on possible variations in the stability due to normal aging. In general, both young and older subjects showed similar overall learning rates of adaptation and by the end of training there was not a significant difference in the level of explicit aiming and implicit learning between the two groups. Interestingly, over a short-term delay period (from 0s to 90s), both young and older subjects showed almost complete retention of the explicit aiming achieved during adaptation. However, the temporal change for implicit learning was very different between subject groups. Consistent with previous work, young subjects had an exponential decrease in the implicit learning component. In stark contrast, older subjects had a linear decrease over the same time period. These results demonstrate that within the context of this motor adaptation task, normal

aging reduces the short-term stability of implicit motor learning across time, with very little impact

on explicit motor learning processes.”

2. Please add titles to the panels of Figures in addition to panel labels. It would be much easier to understand what is presented in each panel.

As suggested, in the revised manuscript we have added panel titles to all figures.

3. It would be more correct to call ‘elderly’ group as ‘older’ group

We agree with this suggestion. Throughout the revised manuscript we replaced the term ‘elderly’

with ‘older’.

4. How does Fig.5 contribute to our understanding of possible differences in short-term retention between young and old adults. Furthermore, I don’t agree that this is consistent with Haith et al. (lines 389-391) given that their low and high PT was in the order of a few 100ms and not several seconds. In addition, it does not make sense that the slope was steeper for older adults early in the adaptation period.

The Reviewers are correct and we apologize for the mistake. The slope for the older adults was less steep compared to the young subjects in the early adaption period. In the revised manuscript we now correct this mistake and soften our comparison to the previous work of Haith et al. and provide more context (multiple timescales of movement planning) of why this analysis gives novel insight (Lines 642-667):

"Our results presented in Figure 5 suggests that during early training the increase of the explicit aiming was related with the increase of the selection time; with longer selection time subjects in both groups had a larger explicit aim to counter the perturbation. Although demonstrated on a significantly smaller time scale (milliseconds as opposed to seconds/minute), this benefit of increased decision time is observed in previous findings (Haith et al. 2015, 2016) where subjects exhibited significantly greater error on trials with low preparation time compared with high preparation time trials. Importantly, the results suggest that the time allowed to prepare the motor output differentially influence the explicit an d implicit learning components-more time allows a greater contribution of explicit strategy. The shallower relationship in early training for older subjects suggests that they required more time to form an aiming direction that young subjects were able to formulate in much less time. In addition, all subjects required more time to select an aiming direction as the delay period increased (Figure 8). However, similar to training (Figure 4), the older subjects often required twice the amount of time compared to young subjects, suggesting greater uncertainty in forming the explicit plan. This finding is consistent with prior studies (Trewartha et al. 2014; 2016), where older subjects had a longer task reaction time during an explicit memory task. Furthermore, the increased reaction time in the older group for the memory task was found to correspond with reduced explicit memory performance, demonstrating a negative relationship between reaction time and explicit memory. Trewartha (2016) demonstrated similar findings in the context of a weight prediction task. Our results did not show a similar temporal relationship; older subjects were able to maintain aiming accuracy although the selection time increased (Figures 7 and 8). However, in both the training and testing phase, the longer selection time for older subjects may result from uncertainty in the aiming direction, rather than a general difficulty in making the selection. That is, there was no significant change in the explicit aiming at the end of training (Figure 3) or over the testing phase (Figure 7), but older subjects continued to require significantly more selection time compared to young subjects.”

5. Vandevoorde and Orban de Xivry 2019 reported no difference in short-term retention between young and old participants over a one-minute break for total adaptation and for implicit adaptation only (isolated by design)

We thank the Reviewers for this comment. In the revised manuscript we now clearly contrast the previous finding from Vandevoorde and Orban de Xivry (2019) to the current study (Lines 609-

619):

"It is important to note that these results point to an age-related deficit in the short-term temporal stability, which appears in contrast to the previous finding that older and younger subjects had similar retention of implicit and overall adaptation over a one-minute break during learning (Vandevoorde and de Xivry, 2019). However, Vandevoorde and de Xivry (2019) assessed this retention after a single time point, whereas we were interested in how this retention systematic changed over time. In addition, even in our study this single time point shows similar levels of decay between the two groups (see Figure 7 near the 50 second time point); extending the range beyond a minute (> 90 seconds) provides a full view of the difference in the decay rates. Furthermore, we assessed age-based difference in stability after asymptotic levels of learning had been achieved and maintained (through re-training), not during training as in this previous study.”

6. Why does the effect of group reported on line 429 does not match the effect of group on line

476? The model is different but should not the statistical results be the same because total

adaptation = exp.

We agree with the Reviewers and apologize for the confusion. We revised the entire analysis based on the suggestions detailed above and now use the actual elapsed time as a covariate in our model. In the revised manuscript these new results are presented in the Results section (please see above).
